# Detecting implicit cross-communities to which an active user belongs

**DOI:** 10.1371/journal.pone.0264771

**Published:** 2022-04-19

**Authors:** Kamal Taha, Paul Yoo, Fatima Zohra Eddinari

**Affiliations:** 1 Department of Electrical and Computer Science, Khalifa University, Abu Dhabi, United Arab Emirates; 2 Department of Computer Science & Information Systems, University of London, Birkbeck College, London, United Kingdom; 3 Department of Sociology, University of Texas at Arlington, Arlington, Texas, United States of America; Federal University of Pernambuco: Universidade Federal de Pernambuco, BRAZIL

## Abstract

Most realistic social communities are *multi-profiled cross-communities* constructed from users sharing commonalities that include adaptive social profile ingredients (i.e., natural adaptation to certain social traits). The most important types of such cross-communities are the *densest* holonic ones, because they exhibit many interesting properties. For example, such a cross-community can represent a portion of users, who share *all* the following traits: ethnicity, religion, neighbourhood, and age-range. The denser a multi-profiled cross-community is, the more granular and holonic it is and the greater the number of its members, whose interests are exhibited in the common interests of the entire cross-community. Moreover, the denser a cross-community is, the more specific and distinguishable its interests are (e.g., more distinguishable from other cross-communities). Unfortunately, methods that advocate the detection of granular multi-profiled cross-communities have been under-researched. Most current methods detect multi-profiled communities without consideration to their granularities. To overcome this, we introduce in this paper a novel methodology for detecting the smallest and most granular multi-profiled cross-community, to which an active user belongs. The methodology is implemented in a system called ID_CC. To improve the accuracy of detecting such cross-communities, we first uncover missing links in social networks. It is imperative for uncovering such missing links because they may contain valuable information (social characteristics commonalities, cross-memberships, etc.). We evaluated ID_CC by comparing it experimentally with eight methods. The results of the experiments revealed marked improvement.

## Introduction

A massive number of complex scientific problems have been depicted and represented as network structures for empirical studies. These network representations solve many different scientific fields, such as biological systems [1], ecosystems [2], information systems [3], and scientific citations [4]. Among them, social media ecosystem problems are the most ones delineated using network representation for uncovering community structures. The structure of a society can be well analyzed and studied by clustering its members into communities based on a certain criterion. Such community-based clustering can uncover social groups of various traits such as ethnicity, religion, colleague, research groups, social media collaborators, and family-based.

The methods that cluster data based on its attributes can be broadly classified into the following: (1) methods that use the structural relationships between nodes (e.g., linkage information) as guidance of the clustering procedure [5]; (2) methods that use the information of nodes’ attributes as guidance for clustering [6] (but these methods disregard crucial information pertaining the structural relationships between the nodes), and (3) methods that use both the structural relationships between nodes and the information of node attributes as guidance for clustering [7] (these methods perform clustering based on the similarity of attributes and the density of connectivity). Most of the methods that perform clustering based on the structural relationships between nodes use probabilistic generative models to determine the posterior userships of communities [8, 9].

### Detecting communities from heterogeneous information networks

Most of the above-mentioned methods can detect real-world communities according to specific properties; yet many of them can detect only heterogeneous communities and communities with certain topological structures [10, 11]. To overcome this limitation, other methods have been proposed for detecting communities from heterogeneous information networks [12]. Most real-world applications require the interaction between multi-typed objects. These are heterogeneous information networks (HIN) [13] that have different types of edges and vertices. As an example, a bibliographic network links published papers to various types of objects such as authors, topics, conferences, and journals. Thus, a HIN contains vertices with different types and links representing the relationships between these vertices. These networks possess rich semantic information revealed by their vertices and links.

Ahn et al. [14] proposed a method that regards a community as a set of links rather than a set of nodes to better uncover the hierarchical relationships among different communities. By considering each link as a single context, the method constructs a dendrogram, whose branches represent link communities. Overlapping communities are identified by cutting the dendrogram at various thresholds. To cluster links, the authors introduced a partition density objective function based on link density. Psorakis et al. [15] proposed a probabilistic method that adopts Bayesian non-negative matrix factorization model to extract overlapping community partitions from a single interaction network. The method is based on the assumption that if two nodes belong to a same community, there is a high probability of a link connecting the two nodes. Therefore, the method works better in dense and fully connected subgraphs.

Palla et al. [16] proposed a method that detects overlapping communities by analysing the statistical features of the communities. The method first detects all *k*-cliques in a network and then identifies communities and their overlaps by carrying out clique-clique overlap distribution analysis using the following four quantities: (1) the number of communities, to which each node belongs, (2) the number of nodes shared by two communities, (3) the number of links in a community, and (4) the number of nodes in a community.

### Detecting communities from heterogeneous information networks using the information of their attributed nodes

Many methods that combine clustering analysis and attribute information have been proposed for detecting communities using node attribute information. Aggarwa et al. [17] proposed a method that employs local succinctness property for detecting balanced communities from heterogeneous networks. The authors proved that employing local behaviour is superior to global viewing for detecting communities. The authors attributed this to the difficulty of the variation of local density for building community detection techniques. They investigated social networks’ locality characteristics and constructed an algorithm based on local behaviour. Sun et al. [18] introduced a framework for overcoming the problem of incomplete nodes’ attributes information in heterogeneous information networks. The main contribution of the authors is the development of a probabilistic clustering framework to be used for modelling different types of semantic links in heterogeneous information networks that exhibit incomplete attributes. Qi et al. [19] proposed a method that employs heterogeneous random fields for integrating the structure and content of a network that delineates social media with outlier links. The main contribution of the authors is combining social cues and linkage information after discarding each abnormality in the linkages to enhance the consistency of clustering social media elements. Cruz et al. [20] proposed a method for detecting the community structure in attributed networks. The main contribution of the authors is the integration of the two dimensions in attributed graphs: the compositional dimension (which describes actors) and the structural dimension (which embodies the social graph).

### Detecting cross-communities from heterogeneous information networks using the information of their attributed nodes

Most realistic social communities are multi-attributed cross-communities constructed from users sharing some commonalities. The challenge is how to detect a multi-attributed cross-community from a multi-attributed network, whose some of its attributes are unlabelled. Detecting such a cross-community requires constructing community-specific modelling techniques capable to infer the distinguishable characteristics of each cross-community. The techniques should include mechanisms able at identifying the distinguishable characteristics of each attribute. As can be seen from the current methods described previously that they are not equipped with such mechanisms, except for, to some degree, CoRel [21]. Even CoRel has the drawback of requiring a community’s seed of taxonomy to be given beforehand.

### Our proposed approach

Most current methods detect multi-profiled communities without consideration to their granularities. To overcome this, we introduce in this paper a novel methodology for detecting the smallest and most granular multi-profiled cross-communities. We implemented the methodology in a working system called **I**mplicit **D**etector of **C**ross-**C**ommunities (**ID**_**CC**). ID_CC detects a cross-community at the granularity of a *k*-clique. Current methods that adopt the *k*-clique approach for detecting the cross-community to which an active user belongs (such as [16]), employ the *k*-clique procedure for extracting cross-nodes at the granularity of a cross-communities (as opposed to a cross-*k*-cliques). Detecting cross-*k*-cliques requires quantifying the extent to which each pair of *k*-cliques are associated. To the best of our knowledge, our method is the first that perform the following:

- Quantifying the extent to which *k*-cliques are associated. Current methods simply use the structural positioning of *k*-cliques in a network to assess their relationships. For example, Palla et al. [16] used clique-clique statistical interaction for assessing their relationship (simply the number of their overlapped nodes). On the other hand, ID_CC quantifies clique-clique interaction’s degree of influence in associating their overlapped communities as well as the other communities in the network. The quantification is expressed in terms of scores that serve as indicators of the *local* influence of the pair of cliques’ interaction in associating their overlapped communities and the *global* influence of the pair in associating the other communities in the network. That is, to extract a cross-communities at the granularity of a *k*-clique, ID_CC quantifies the following:
  1. The pair of clique’s binary influence, which is the extent to which the pair’s interaction influences the relationship between their overlapped committees.
  2. The pair of cliques’ global influence, which is the extent to which the pair’s interaction influences the relationships among all communities in the network.
  Let *C*_*i*_ and *C*_*j*_ be two interrelated cliques residing in communities *CMTY*_*x*_ and *CMTY*_*y*_ respectively. Let *CMTY*_*z*_ be an arbitrary community in the network that is not overlapped with *CMTY*_*x*_ and *CMTY*_*y*_. ID_CC quantifies the *influence* of the interaction between *C*_*i*_ and *C*_*j*_ in transmitting information between *CMTY*_*x*_ and *CMTY*_*y*_ (i.e., local influence) and in transmitting information between *CMTY*_*x*_ and *CMTY*_*z*_ and between *CMTY*_*y*_ and *CMTY*_*z*_ (i.e., global influence).
- Inferring missing links prior to detecting cross-communities using novel mechanisms.
- Employing a novel mechanism that can implicitly infer an active user’s undeclared communities that match his own social traits.

Since there are always new users wishing to join existing cross-communities, we incorporated a functionality to ID_CC that detects the smallest and most granular multi-profiled cross-community, to which an active user belongs. First, the system infers the comprehensive list of the user’s communities based on a few communities, to which the user declared membership. That is, the system implicitly infers the user’s undeclared communities that match his own social traits. Then, the system infers the smallest and most granular multi-profiled cross-community, to which the user belongs by analysing the hierarchical interrelationships between the detected user’s communities. The system considers all cross-profiles that come to existence from the interrelationships between the hierarchically overlapped user’s communities. The larger the number of inferred user’s communities, the denser and more specific is the multi-profiled cross-community identified by the system for the user.

Our methodology is based on the following observations, which shed the light on the importance of detecting granular multi-profiled cross-communities and missing links:

1. A community is a social entity with specific social rules and dynamicity commonalities. We observe that most realistic social communities are *multi-profiled cross-communities* constructed from users sharing commonalities that include adaptive social profile ingredients. A community defined by the commonality of its adaptive social profile is a one constructed according to the natural adaptation to a certain social trait as opposed to being constructed due to involuntary circumstances (e.g., a collegial work group). The interests of a multi-profiled cross-community are the union of the interests of the various communities, from which the cross-community is constructed.
2. The most important types of multi-profiled cross-communities are the *densest* holonic ones, because they exhibit many interesting properties. For example, such a cross-community can represent a portion of users, who share *all* the following traits: ethnicity, religion, neighbourhood, and age-range. The denser a multi-profiled cross-community is, the more granular and holonic it is and the greater the number of its members, whose interests are exhibited in the common interests of the entire cross-community. The likelihood of an exact match between the interests of an active user and the interests of his cross-community increases as the cross-community becomes denser. The denser such a cross-community is, the more specific and distinguishable its interests are from other cross communities.
3. The links in most social networks are not exhaustive. The sharing of some social characteristics between a pair of communities may not always be reflected by a link connecting the pair in the social network. Most often, this happens when a social network depicts a dataset containing some members, who did not fully disclose and declare their memberships to all the communities, to which they belong (i.e., did not disclose all their cross-memberships). It is imperative for uncovering such missing links because they may contain valuable information (social characteristics commonalities, cross-memberships, etc.). Therefore, detecting cross-communities should be preceded by uncovering missing links.
4. Methods that advocate the detection of granular multi-profiled cross-communities have been under-researched. Most these methods detect cross-communities without consideration to their granularities.

## Concepts used in the paper and outline of the approach

### Concept of overlapping multi-attribute community

We use the term “**S**ingle-**A**ttribute **c**ommunity” (**SAC**) throughout the article to refer to an aggregation of individuals who share a common single attribute (e.g., a same ethnicity). This concept is formalized in definition 1.

**Definition 1—Single-Attribute Community (SAC)**: SAC is a group *G* of individuals within a social network *(V*, *E)* with schema *(R*, *L)*, where each *x*, *y ∈ G (x* ≠ *y)* share one common single attribute mapping *ψ*: *V → R* and relation mapping *∂*: *E → L*.

The denser a community is, the more distinct and specific are its common concerns and interests. Therefore, we propose a granular and specific class of community called **M**ulti-**A**ttribute **C**ommunity (**MAC**). A MAC is formed from an aggregation of individuals who all belong to two or more SACs. That is, the common characteristic shared by these individuals are the attributes of several SACs. Thus, a MAC is an aggregation of members who share common *multi-attributed* traits. Intuitively, the size of such a MAC is smaller than the size of each of the SACs, to whom its individuals belong. An **O**verlapping **M**ulti-**A**ttribute **C**ommunity (**OMAC**) is a MAC formed from an aggregation of individuals who all belong to two or more SACs of *different attributes*. That is, an OMAC is a body of members who share the common characteristics of some cross-community’s multiple attributes.

**Fig 1:**
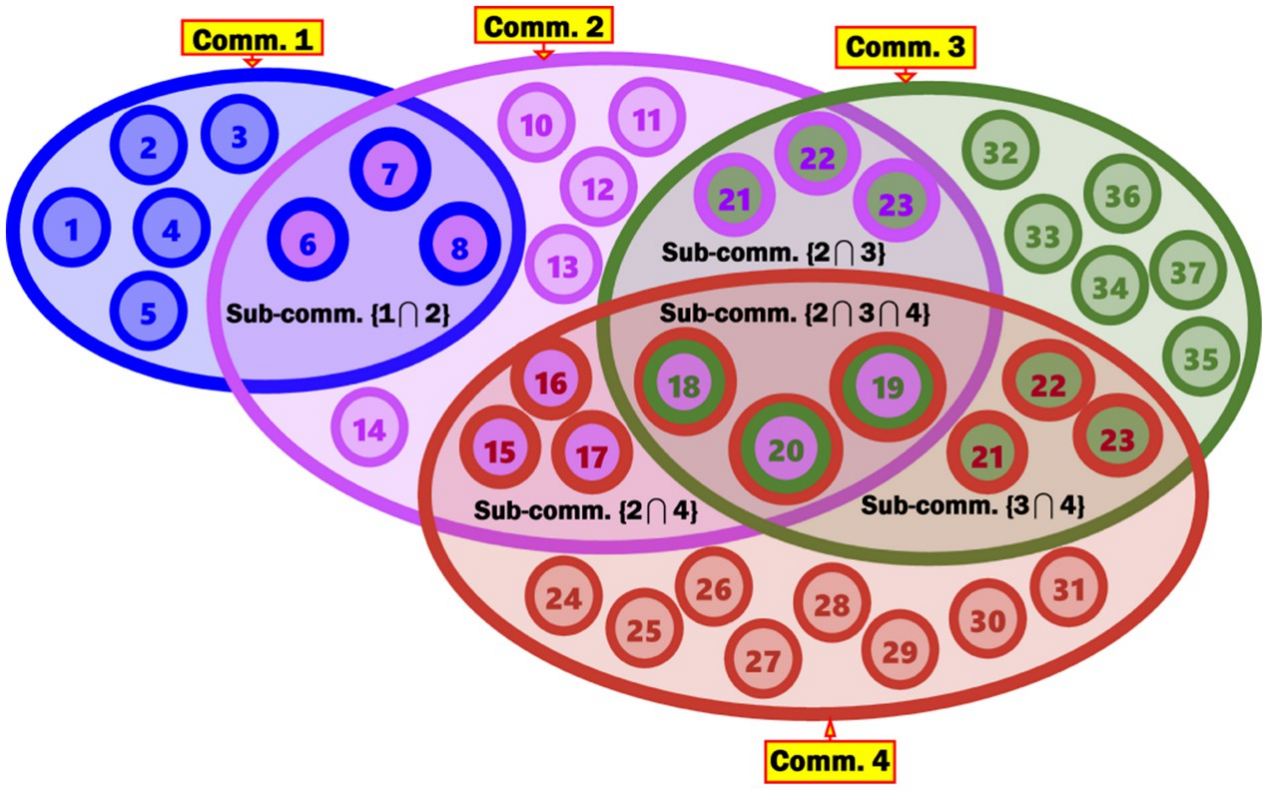
Hypothetical four communities and the subcommunities resulting from the overlapping of these communities.

***Example***: Consider user member 18 in Fig 1. This user belongs to the following communities and subcommunities: 2, 3, 4, {2∩4}, {3∩4}, and {2∩3∩4}. Intuitively, the densest and most granular multi-profiled cross-community, to which user 18 belongs is {2∩3∩4}. Consider that community 2 represents an ethnic group E(*x*), community 3 represents a religion R(*y*), and community 4 represents a national origin O(*z*). Then, the densest and most granular multi-profiled cross-community, to which user 18 belongs will be formed from an aggregation of individuals who belong to the same ethnic group E(*x*), follow the identical religion R(*y*), and are descendants of the matching national origin O(*z*). Thus, such OMAC is constructed from the following intersection: E(*x*) ∩R(*y*) ∩O(*z*). The interests and concerns of this OMAC are more specific and granular than the ones of each of E(*x*), R(*y*), and O(*z*) individually.

### Concept of Maximal *k*-Clique Sub-SAC

An effective mechanism for identifying the influential nodes in a SAC is to first represent the SAC using *k*-clique model. A *k*-clique is a defined as complete graph with *k* nodes. A pair of adjacent *k*-cliques shares *k*-1 nodes. We now formalize these concepts.

*Definition 1—Clique*: A clique *C* in a graph *G* is a subset of the nodes of *G* such that every two nodes in *C* are adjacent. Thus, *C* is a complete induced subgraph.

*Definition 2—k-Clique*: It is a clique of a subcommunity that has *k* nodes. Each two adjacent *k*-cliques *C*_*i*_ and *C*_*j*_ in the subcommunity share *k* − 1 nodes. That is, *C*_*i*_ ∩ *C*_*j*_ = *k* − 1.

We introduce the concept of **M**aximal ***k***-**C**lique **S**ub-**S**AC (**MKCSS**). A MKCSS is a subcommunity within a SAC formed from the maximal union of *k*-cliques within the SAC, where each two *k*-cliques in the subcommunity is *k*-clique connected. It is a fully connected (complete) subcommunity of *k*-cliques within a SAC. We now formalize these concepts.

*Definition 3—Maximal k-Clique Sub-SAC (MKCSS)*: It is a maximal union of *k*-cliques within a SAC, where each two *k*-cliques *C*_*i*_ and *C*_*j*_ in the subcommunity is *k*-clique connected. *C*_*i*_ and *C*_*j*_ are *k*-clique connected, if there is a series of *k*-cliques *C*_*x*_, *…*, *C*_*y*_, such that each two adjacent cliques in the series *C*_*i*_, *C*_*x*_, *…*, *C*_*y*_, *C*_*j*_ share *k* -1 nodes.

**Lemma 1**: Any two *k*-cliques in a MKCSS are *k*-clique connected.

**Proof**: If we consider a scenario of |MKCSS| = *k* + 1, any two *k*-cliques $C_{1}^{k}$ and $C_{2}^{k}$ in the MKCSS share *k* + 1 nodes. This is because $C_{1}^{k}\cap C_{2}^{k}=k+1$. Similarly, if we consider a scenario of |MKCSS| = *k* + 2, any two *k*-cliques $C_{1}^{k+1}$ and $C_{2}^{k+1}$ in the MKCSS share *k* + 1. Since: (a) $C_{1}^{k}$ is connected to any *k*-clique in $C_{1}^{k+1}$, and (b) $C_{2}^{k}$ is connected to any *k*-clique in $C_{1}^{k+1}$, $C_{1}^{k}$ and $C_{2}^{k}$ are *k*-clique connected. The above holds for any |MKCSS| > *k*.

**Fig 2:**
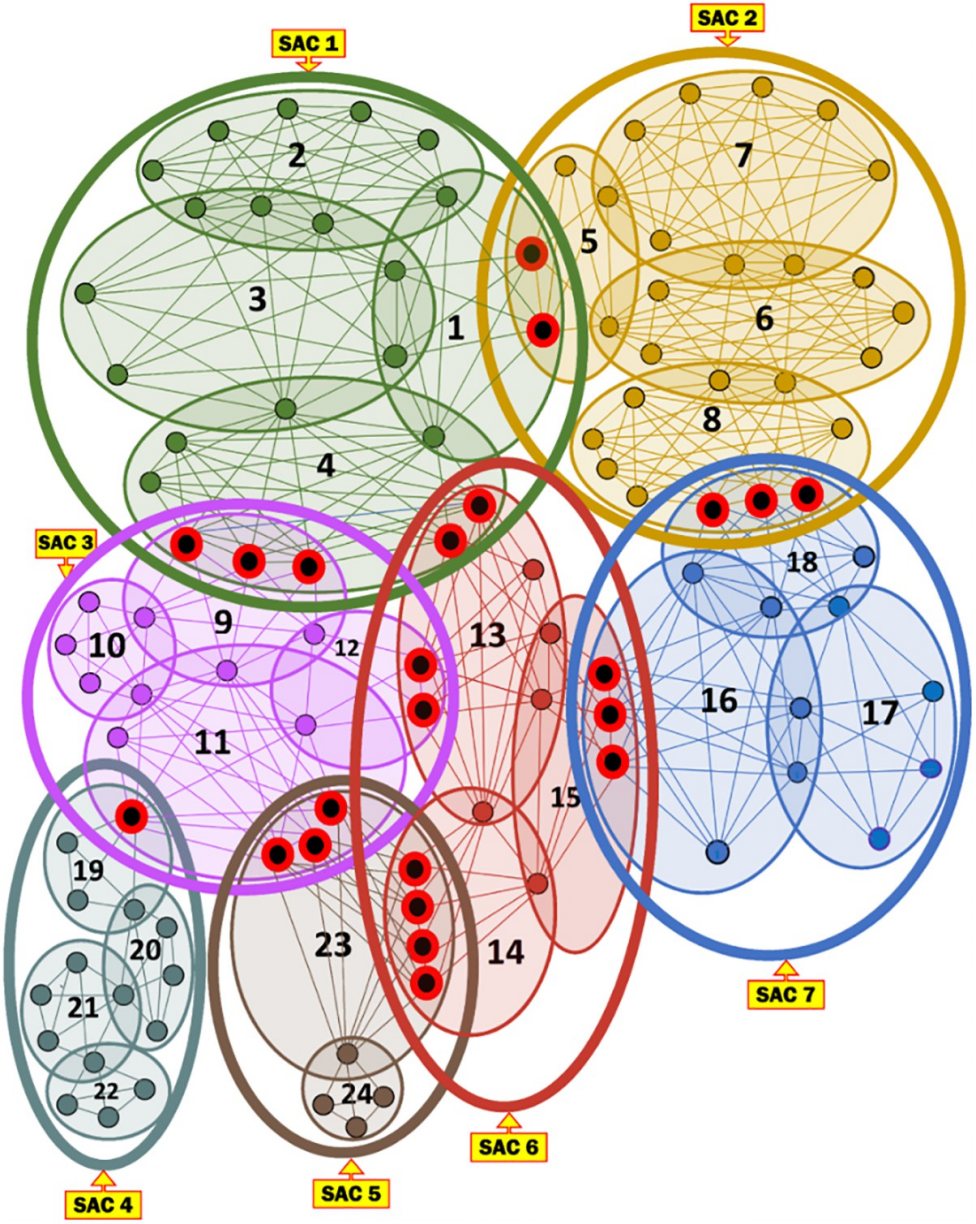
Seven illustrative SACs along with their MKCSSs, which we will be using as a running example throughout the paper. The MKCSSs are constructed based on the 4-clique modelling.

***Running Example 1***: Fig 2 depicts the MKCSSs of seven SACs, representing some social media messaging. The MKCSSs are constructed based on the 4-clique modelling. Each SAC contains a number of MKCSSs. For example, SAC 1 consists of MKCSSs 1–4. Some MKCSSs share cross-members (marked in red with black centre).

### Concept of MKCSS Relationship Graph

We now introduce the concept of **M**KCSS **R**elationship **G**raph (MRG), which depicts the relationships between the MKCSSs of a same SAC as well the interrelationships between the MKCSSs of different SACs. Each node in the MRG represents a MKCSS. Two nodes in the MRG are connected by an edge if they share at least one cross-member. Thus, two SACs in the MRG are connected by an edge if they share at least one cross-member. We now formalize this concept.

*Definition 4*: *MKCSS Relationship Graph (MRG)*: An MRG is an undirected graph *G* (*V*, *E*), where *V* is the set of nodes and *E* is the set of edges in the graph. Each node in *G* represents an MKCSS in some SAC. The weight of an MKCSS node is the number of unique *k*-cliques in the MKCSS. Two nodes *n*_*i*_, *n*_*j*_ ∈ *V* are connected by an edge *e* ∈ *E*, if *n*_*i*_ and *n*_*j*_ share at least one common individual member (i.e., cross-member).

**Theorem 1**: Since the weight of a MKCSS node is the number of its unique *k*-cliques, the weight of the MKCSS node equals |MKCSS|! (k! (|MKCSS| − k)!)

**Proof**: The number of unique *k*-cliques in an MKCSS is the number of unique *k*-combination of a subset of *k* distinct nodes that belong to the MKCSS. The number of these *k*-combinations equals the binomial coefficient, which can be depicted using factorials as follows: |MKCSS|! (k! (|MKCSS| − k)!).

**Fig 3:**
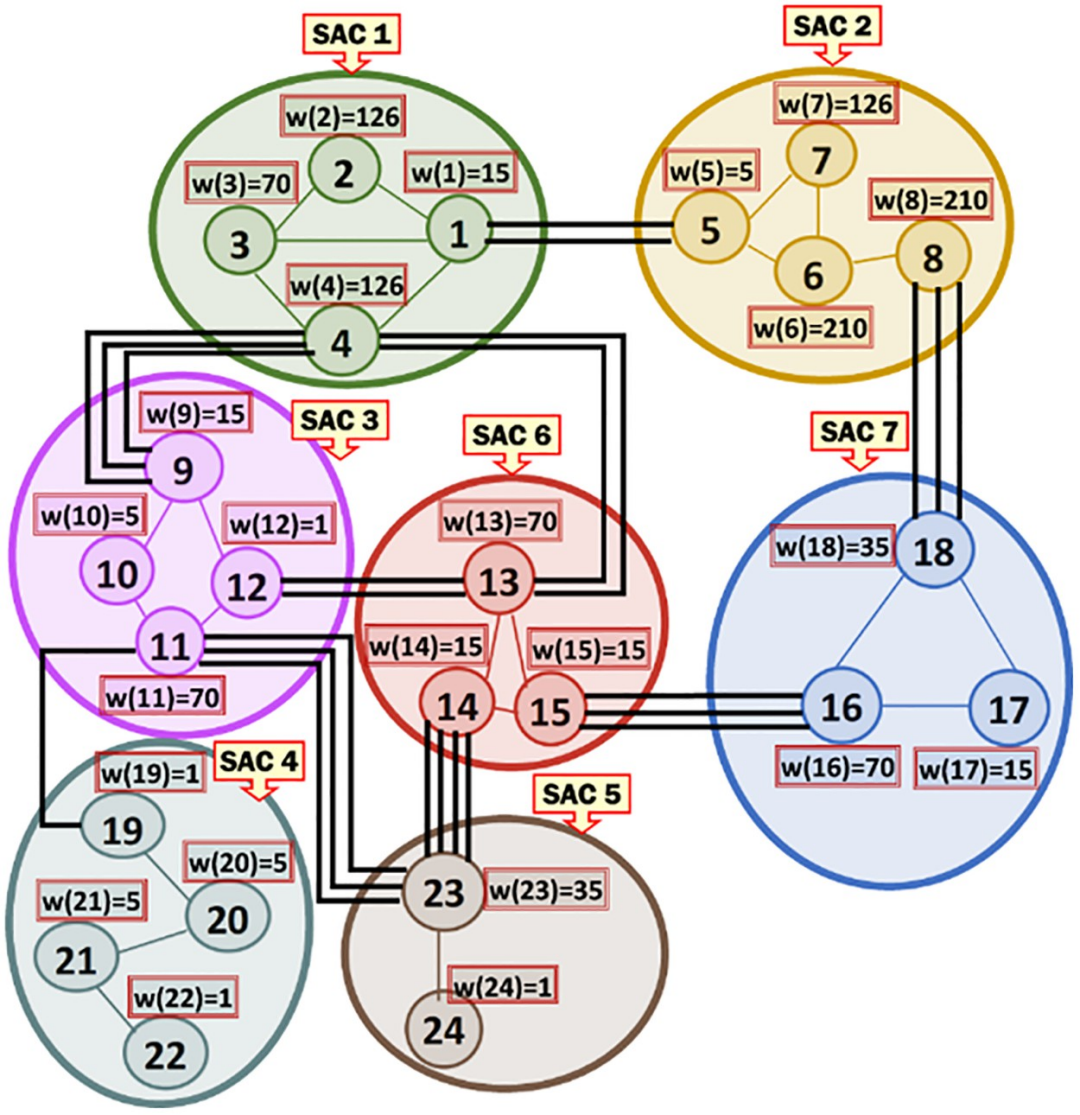
The MRG that corresponds to the MKCSSs in Fig 2. Node *i* in Fig 3 represents MKCSS *i* in Fig 2.

***Running Example 2***: Fig 3 shows the MRG that corresponds to the SACs and MKCSSs shown in Fig 2. Node *i* in Fig 3 represents MKCSS *i* in Fig 2. For example, node 1 in Fig 3 represents MKCSS 1 in Fig 2. The weight of node *i* (i.e., w(*i*)) in Fig 3 is the numbers of 4-cliques inside node *i* in Fig 2. For example, the weight of node 1 in Fig 3 is 15 (i.e., w(1) = 15). An edge connecting two nodes in Fig 3 signifies that the two nodes share at least one user (i.e., cross-member) in the corresponding MKCSSs in Fig 2.

### Concept of Association Edge

An Association Edge denotes cross-members shared by two interrelated MKCSSs that belong to two different SACs. Let M_i_ and M_j_ be two MKCSSs that belong to two SACs and share cross-members. M_i_ and M_j_ in the MRG will be linked by an Association Edge to denote that they share cross-members. For example, there are three Association Edges connecting SACs 2 and 7 in the MRG in Fig 3 due to cross-members shared by MKCSSs 8 and 18 (recall Fig 2).

### Concept of Binary Influence

A Binary Influence (BI) is a score that quantifies the degree of influence of an Association Edge in associating the two SACs at its end points. That is, the score quantifies the extent to which the cross-members shared by two SACs relate the two SACs. Thus, BI is an indicator of the *local* influence of an Association Edge.

### Concept of Global Influence

A Global Influence (GI) is a score that quantifies the degree of influence of an Association Edge in associating all SACs in MRG. That is, the score characterizes an Association Edge’s *global* influence in the entire MRG. Thus, GI reflects the global relative interaction role and influence of an Association Edge in passing information to the entire network.

For convenient reference, we list in Table 1 abbreviations of the major terms that appear in the article.

**Table 1:** Abbreviations of the concepts presented in the article.

*Abbreviation*	*Description*
**SAC**	Single-Attribute Community
**OMAC**	Overlapping Multi-Attribute Community
**MKCSS**	Maximal k-Clique Sub-SAC
**MRG**	MKCSS Relationship Graph
**Association Edge**	An edge that depicts the cross-users of two interrelated cross-MKCSSs
**BI (Binary Influence)**	A score quantifies the degree of an Association Edge’s influence in associating the two SACs
**GI (Global Influence)**	A score quantifies the degree of an Association Edge’s influence in associating all the SACs
**RLCA**	Relevant Lowest Common Ancestor

### Outline of the approach

Below are the sequential processing steps taken by our proposed system ID_CC:

1. ***Computing the Binary Influence of each Association Edge***: ID_CC computes the *BI*(*μ*, *ν*) score of each Association Edge (*μ*, *ν*) connecting a pair of SCAs *S*_*μ*_ and *S*_*ν*_ in MRG. The score reflects the local influence of (*μ*, *ν*) relative to the other Association Edges connecting *S*_*μ*_ and *S*_*ν*_.
2. ***Computing the Global Influence of each Association Edge***: ID_CC computes the *GI*(*μ*, *ν*) score of each Association Edge (*μ*, *ν*) connecting a pair of SCAs *S*_*μ*_ and *S*_*ν*_ in the MRG. The score reflects the average chance of (*μ*, *ν*) relative to the other Association Edges connecting *S*_*μ*_ and *S*_*ν*_ in passing information between: (a) *S*_*μ*_ and *S*_*ν*_, and (b) the remaining SACs in the MRG. The formula for computing *GI*(*μ*, *ν*) considers various factors such as the BI of (*μ*, *ν*) as a fraction of the BIs of all Association Edges that pass information between: (a) *S*_*μ*_ or *S*_*ν*_, and (b) the remaining SACs in the MRG.
3. ***Uncovering Missing Association Edges in the MKCSS Relationship Tree***: First, ID_CC converts the MRG into a tree data structure for the ease of uncovering missing Association Edges connecting hierarchical interrelated SACs. We call the resulting structure MKCSS Relation Tree. Then, ID_CC uncovers the missing Association Edges using a concept that we call Relevant Lowest Common Ancestor (RLCA), which helps in inferring related nodes based on the relationships between their ancestor nodes. Finally, ID_CC computes the BIs and GIs of the implicitly identified Association Edges.
4. ***Determining the Densest Multi-Profiled Cross-SACs to which an Active User Belongs***: ID_CC applies the Maximum Spanning Tree (MaxST) algorithm [22, 23] on the revised MKCSS Relationship Tree (i.e., the one that includes the implicitly identified Association Edges). All SAC nodes located in the path of the MaxST that connects the user’s revealed (i.e., already known) SAC nodes, will be considered the comprehensive list of SACs, to which the user belongs. The extra SACs in the list (excluding the ones declared by the user) are *implicitly* identified. The resulting intersection of all SACs in the list is the densest and most granular multi-profiled cross-SACs that matches the user’s own social traits.

### Computing the Binary Influence of each Association Edge

**Fig 4:**
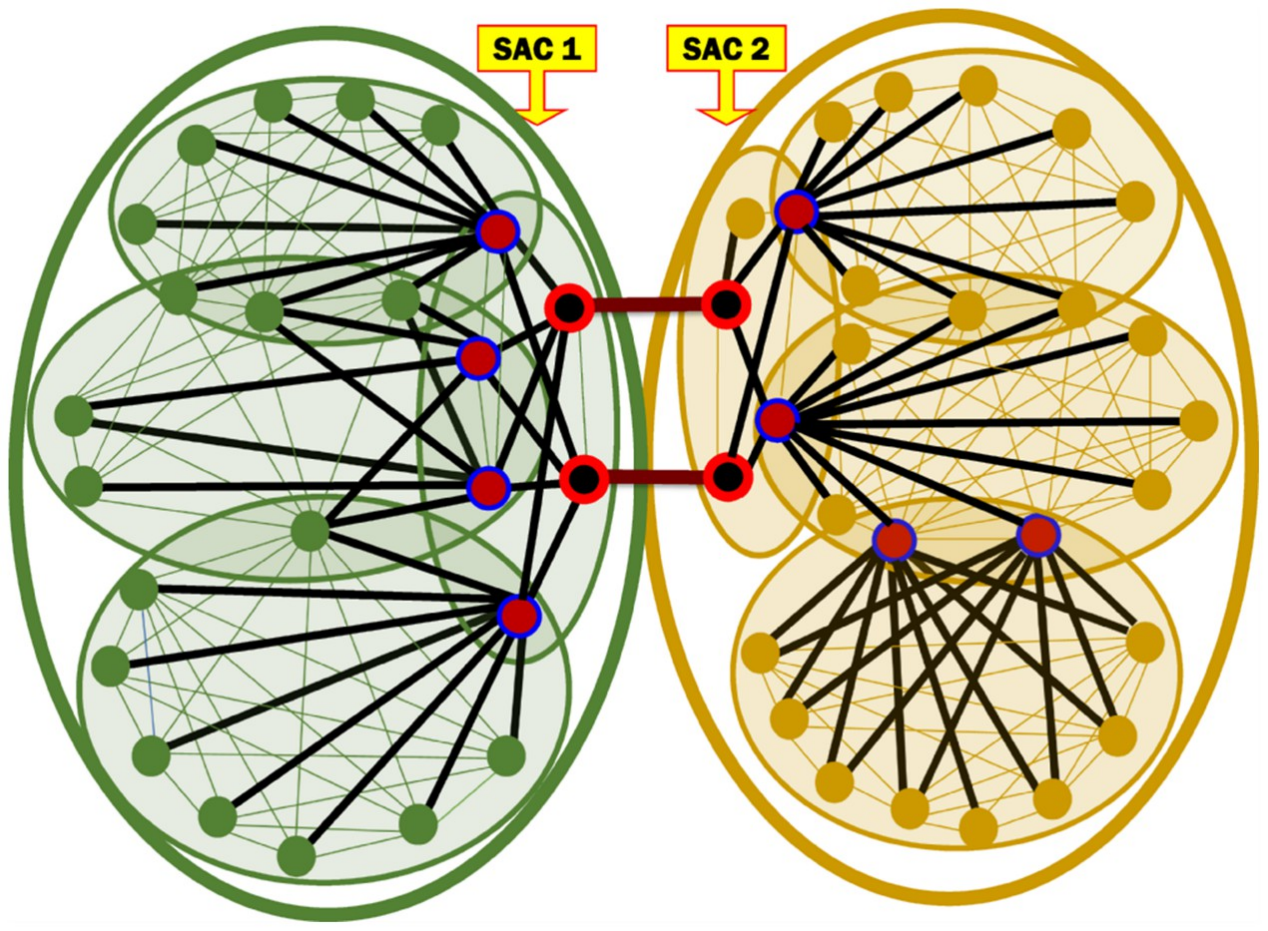
An excerpt from Fig 2 shows SACs 1 and 2 and their two Association Edges to illustrate the factors that impact the BI score.

We describe in this section a mechanism we developed for quantifying the Binary Influence (BI) of an Association Edge. That is, we quantify the extent to which the cross-members shared by two SACs relate the two SACs. The quantification is expressed in terms of a score that serves as an indicator of the *local* influence of the Association Edge [24]. The BI score of an Association Edge reflects the edge’s chance of passing information between the two SACs at its end points relative to the other Association Edges connecting the same two SACs. Consider Fig 4, which is an excerpt from Fig 2 and shows SACs 1 and 2 and the two Association Edges connecting them. Let *ę* denote one of these two edges. As demonstrated by Fig 4, the chance of *ę* to pass a message between the two SACs is impacted by the following factors:

1. Number of edges in the shortest path connecting the two communicating MKCSSs and includes *ę*. Consider that some node *n* belongs to SAC 1 needs to send a message to some node *ń* residing in SAC 2. From the two Association Edges, intuitively, the message will be sent through the one, whose path from *n* to *ń* is the shortest (containing the *smaller number of edges*).
2. Number of member users, who belong to all the MKCSSs located in the shortest path that includes *ę*.
3. Number of Association Edges (i.e., number of cross-members) connecting the two communicating MKCSSs.

**Fig 5:**
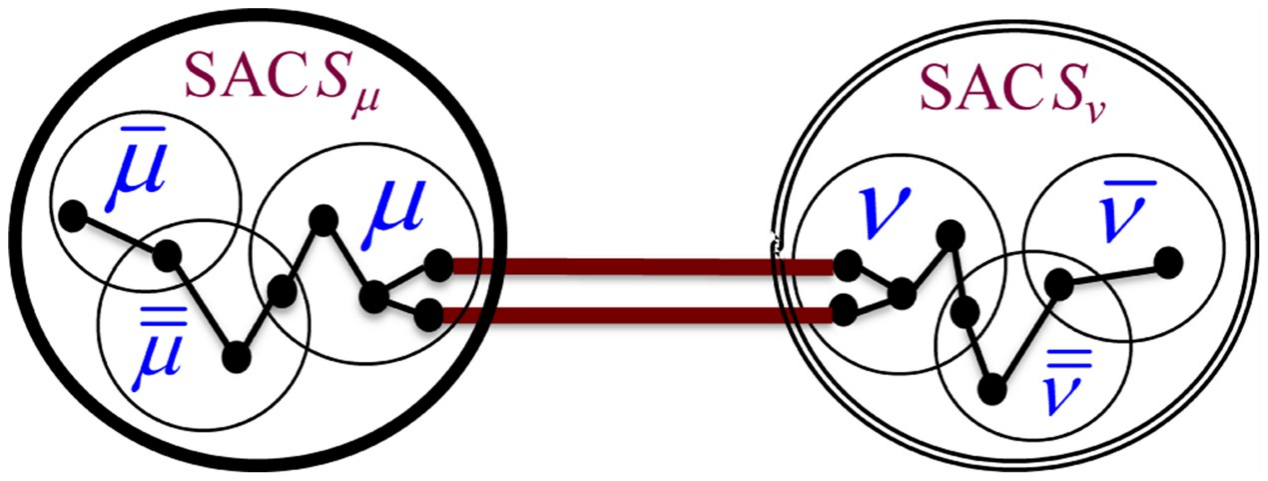
Generic notations used for constructing the formula in Eq 1 for computing the *BI*(*u*, *v*) of an Association Edge (*μ*, *ν*). The figure depicts a generic pair of communicating MKCSSs $\bar{\mu }$ and $\bar{\nu }$ that belong to SACs *S*_*μ*_ and *S*_*ν*_, respectively, whose messages pass through (*μ*, *ν*).

Based on the above observations, we constructed the formula in Eq 1 for computing the BI score of an Association Edge (*μ*, *ν*) connecting two SACs *S*_*μ*_ and *S*_*ν*_. The equation quantifies the influence of the Association Edge (*μ*, *ν*) in passing information between each pair of nodes, one residing at *S*_*μ*_ and the other at *S*_*ν*_. We constructed the formula based on the generic notations shown in Fig 5, which depicts a generic pair of communicating MKCSSs $\bar{\mu }$ and $\bar{\nu }$ that belong to SACs *S*_*μ*_ and *S*_*ν*_, respectively.


$$BI(\mu ,v)=\sum_{\bar{\mu },\bar{\bar{\mu }}\in \text{SAC}\;S_{\mu }}\left(\sigma (\bar{\mu },\mu )\times \left(\left|\bar{\mu }\right|-\left|\bar{\mu }\cap \bar{\bar{\mu }}\right|\right)\times \left|\bar{\mu }\cap \bar{\bar{\mu }}\right|\right)+\sum_{\bar{\nu },\bar{\bar{\nu }}\in \text{SAC}\;S_{v}}\left(\sigma (\bar{\nu },v)\times \left(\left|\bar{\nu }\right|-\left|\bar{\nu }\cap \bar{\bar{\nu }}\right|\right)\times \left|\bar{\nu }\cap \bar{\bar{\nu }}\right|\right)$$ (1)

- *BI*(*μ*, *ν*): The Binary Influence of Association Edge (*μ*, *ν*), which connects SACs *S*_*μ*_ and *S*_*ν*_.
- *S*_*μ*_: The SAC that contains MKCSS node *μ*.
- *S*_*ν*_: The SAC that contains MKCSS node *ν*.
- $\bar{\mu }$, $\bar{\nu }$: Two communicating MKCSS nodes residing in SACs *S*_*μ*_ and *S*_*ν*_ respectively.
- $\left|\bar{\mu }\right|$, $\left|\bar{\nu }\right|$: Number of nodes in MKCSSs *μ* and *ν* respectively.
- $\bar{\bar{\mu }}$: The MKCSS adjacent to $\bar{\mu }$ that resides in the shortest path between $\bar{\mu }$ and *μ* in *S*_*μ*_.
- $\bar{\bar{\nu }}$: The MKCSS adjacent to $\bar{\nu }$ that resides in the shortest path between $\bar{\nu }$ and *ν* in *S*_*ν*_.
- $\left|\bar{\mu }\cap \bar{\bar{\mu }}\right|$: Number of overlapped nodes between MKCSSs $\bar{\mu }$ and $\bar{\bar{\mu }}$.
- $\sigma (\bar{\mu },\mu )$: Number of edges in the shortest path between $\bar{\mu }$ and *μ*.
- $\sigma (\bar{v},v)$: Number of edges in the shortest path between $\bar{\nu }$ and *ν*.

As demonstrated by Fig 5, there may be more than one user member shared by SACs *S*_*μ*_ and *S*_*ν*_ (i.e., multiple cross-members). Each of them is represented by an Association Edge that controls some of the information flow between the two SACs. Intuitively, the BI of each of these edges is impacted by the number of other Association Edges connecting the two SACs. The influence of the edge increases as the number of other Association Edges decreases. That is, the influence of the edge increases at the expense of the other edges. Let |*μ* ∩ *ν*| be the number of Association Edges connecting SACs *S*_*μ*_ and *S*_*ν*_ (i.e., number of cross-members). We need to adjust Eq 1 to keep assigning a lower BI score to an Association Edge as |*μ* ∩ *ν*| increases. This is because as |*μ* ∩ *ν*| increases, the influence of each edge in controlling the flow of information between *S*_*μ*_ and *S*_*ν*_ decreases. Reversibly, as |*μ* ∩ *ν*| decreases, the influence of each edge increases at the expense of the other edges. We adjusted Eq 1 accordingly as shown in Eq 2. That is, the adjusted formula considers the degree into which each edge controls the flow of information over the network.


$$BI(\mu ,v)=\frac{\sum_{\bar{\mu },\bar{\bar{\mu }}\in \text{SAC}\;S_{\mu }}\left(\sigma (\bar{\mu },\mu )\times \left(\left|\bar{\mu }\right|-\left|\bar{\mu }\cap \bar{\bar{\mu }}\right|\right)\times \left|\bar{\mu }\cap \bar{\bar{\mu }}\right|\right)+\sum_{\bar{\nu },\bar{\bar{\nu }}\in \text{SAC}\;S_{v}}\left(\sigma (\bar{\nu },v)\times \left(\left|\bar{\nu }\right|-\left|\bar{\nu }\cap \bar{\bar{\nu }}\right|\right)\times \left|\bar{\nu }\cap \bar{\bar{\nu }}\right|\right)}{2^{\left|\mu \cap v\right|}}$$ (2)

We adjusted the formula in Eq 2 to take into consideration the eigenvector principle [25] by employing logarithm. The adaptation of logarithm enables the formula to characterize an Association Edge’s *relative* influence in passing information between SACs *S*_*μ*_ and *S*_*ν*_. Specifically, the adaptation of logarithm penalizes and rewards the Association Edge exponentially based on its degree of passing information between the two SACs relative to the other Association Edges. This helps in discriminating between an Association Edge among the following lists of Association Edges: (a) a list with varying very large number of Association Edges, and (b) a list with varying very small number of Association Edges. We adjusted Eq 2 accordingly as shown in Eq 3.


$$BI(\mu ,\nu )=\frac{\sum_{\bar{\mu },\bar{\bar{\mu }}\in \text{SAC}\;S_{\mu }}\left(\sigma (\bar{\mu },\mu )\times \left(\left|\bar{\mu }\right|-\left|\bar{\mu }\cap \bar{\bar{\mu }}\right|\right)\times \left|\bar{\mu }\cap \bar{\bar{\mu }}\right|\right)+\sum_{\bar{\nu },\bar{\bar{v}}\in \text{SAC}\;S_{v}}\left(\sigma (\bar{\nu },v)\times \left(\left|\bar{\nu }\right|-\left|\bar{\nu }\cap \bar{\bar{\nu }}\right|\right)\times \left|\bar{\nu }\cap \bar{\bar{\nu }}\right|\right)}{2^{{\text{log}}_{2}}{}^{\left|\mu \cap v\right|}}$$ (3)

Finally, we need to adjust the formula in Eq 3 to reflect the holistic view of the degree of association between the two SACs *S*_*μ*_ and *S*_*ν*_. Towards this, we aim at quantifying the collective influence of all the Association Edges that connect *S*_*μ*_ and *S*_*ν*_ in passing information between the two SACs. That is, we adjusted the equation in such a way that *BI*(*μ*, *ν*) computes one holistic BI score that reflects the collective Association Edges’ influence in passing information between the two SACs. We adjusted the equation accordingly as shown in Eq 4.


$$BI(\mu ,v)=\left|\mu \cap v\right|\times \left(\frac{\sum_{\bar{\mu },\bar{\bar{\mu }}\in \text{SAC}\;S_{\mu }}\left(\sigma (\bar{\mu },\mu )\times \left(\left|\bar{\mu }\right|-\left|\bar{\mu }\cap \bar{\bar{\mu }}\right|\right)\times \left|\bar{\mu }\cap \bar{\bar{\mu }}\right|\right)+\sum_{\bar{\nu },\bar{\bar{v}}\in \text{SAC}\;S_{v}}\left(\sigma (\bar{\nu },v)\times \left(\left|\bar{\nu }\right|-\left|\bar{\nu }\cap \bar{\bar{\nu }}\right|\right)\times \left|\bar{\nu }\cap \bar{\bar{\nu }}\right|\right)}{2^{{\text{log}}_{2}}{}^{\left|\mu \cap v\right|}}\right)$$ (4)

**Fig 6:**
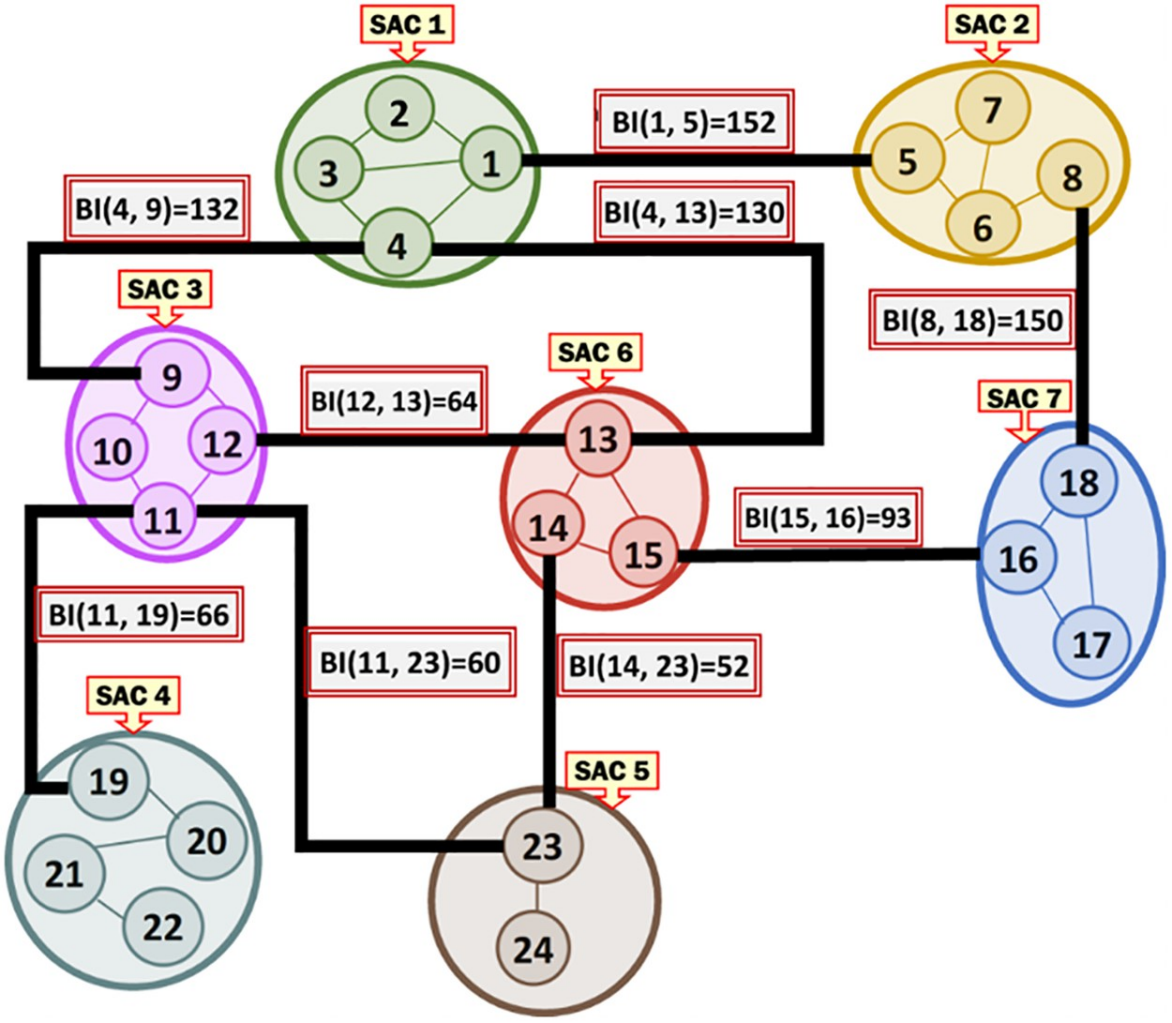
The BI scores of all the Association Edges in our running MRG example after applying Eq 4 (recall Fig 3).

***Running Example 3***: To better illustrate Eq 4‘s composition and components, we show below how the BI score of Association Edge (1, 5) in our running MRG example is computed using Eq 4. Fig 6 shows the BI scores of all the Association Edges in our running MRG example after applying Eq 4 (recall Fig 3).


$$\text{BI}(1,5)=2\times \left(\frac{\;\overset{\text{MKCSS}\;1}{\overset{⎴}{\left(1\times 4\times 2\right)}}+\overset{\text{MKCSS}\;2}{\overset{⎴}{\left(2\times 8\times 1\right)}}+\overset{\text{MKCSS}\;3}{\overset{⎴}{\left(2\times 6\times 2\right)}}+\overset{\text{MKCSS}\;4}{\overset{⎴}{\left(2\times 8\times 1\right)}}+\overset{\text{MKCSS}\;5}{\overset{⎴}{\left(1\times 3\times 2\right)}}+\overset{\text{MKCSS}\;6}{\overset{⎴}{\left(2\times 9\times 1\right)}}+\overset{\text{MKCSS}\;7}{\overset{⎴}{\left(2\times 8\times 1\right)}}+\overset{\text{MKCSS}\;8}{\overset{⎴}{\left(3\times 8\times 2\right)}}\;}{2^{{\text{log}}_{2}2\overset{\left(\text{MKCSS}\;1\cap \text{MKCSS}\;5\right)}{\leftarrow }}}\right)=152$$

### Computing the Global Influence of each Association Edge

**Fig 7:**
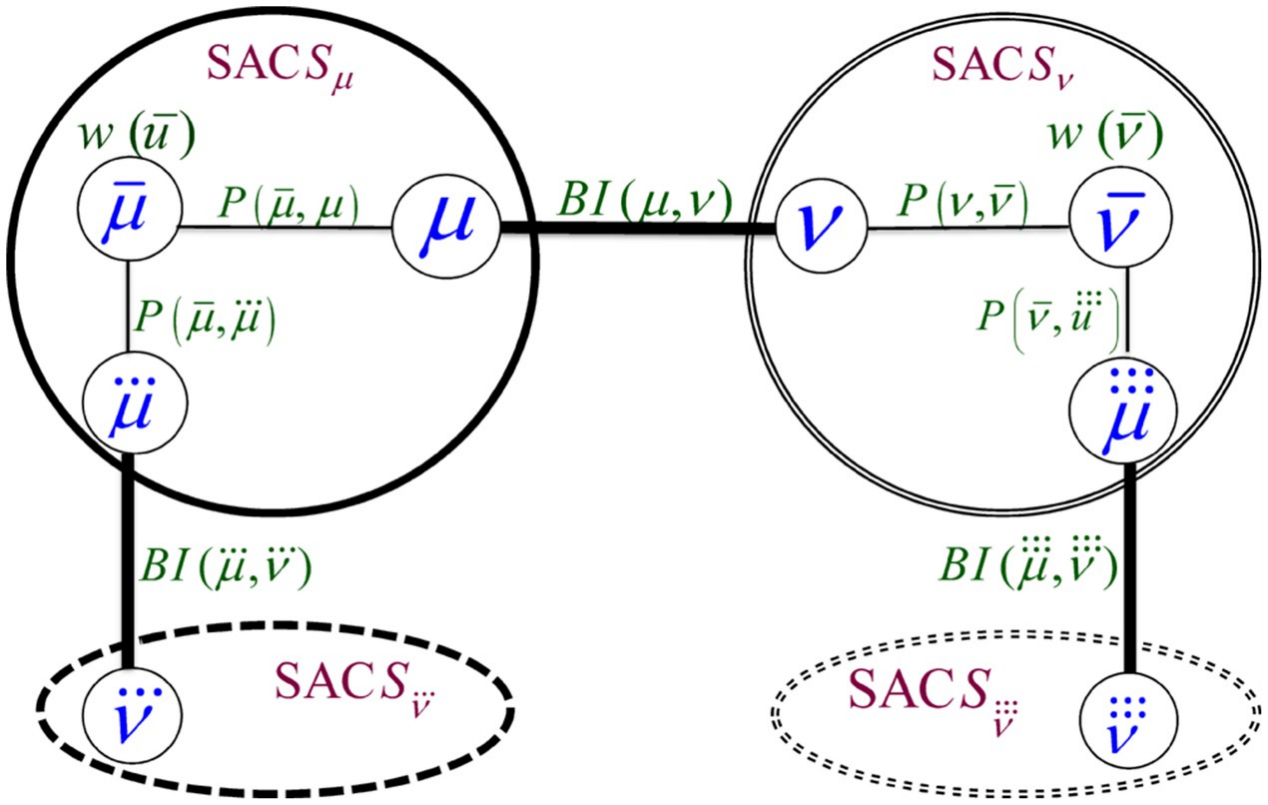
Generic notations used for constructing the formula in Eq 5 for computing the *GI*(*μ*, *ν*) of an Association Edge(*μ*, *ν*).

We heuristically developed a formula that assigns each Association Edge a GI score that characterizes its *global* relative influence in associating all SACs in the MRG. We constructed the formula in such a way that it quantifies the degree of influence of the Association Edge in terms of its average chance in passing information between the SACs at its end points and the remaining SACs in the MRG. That is, the formula computes the average chance of an Association Edge (*μ*, *ν*) connecting two SACs *S*_*μ*_ and *S*_*ν*_ relative to the other Association Edges connecting the two SACs in passing information between: (a) *S*_*μ*_ or *S*_*ν*_, and (b) the remaining SACs in the MRG. The score is computed based on the BI of (*μ*, *ν*) as a fraction of the BIs of all Association Edges that pass information between: (a) *S*_*μ*_ or *S*_*ν*_, and (b) the remaining SACs in the MRG. By applying the above on the generic SACs in Fig 7, we constructed the formula in Eq 5.


$$GI(\mu ,v)=\frac{\left(\sum_{\bar{\mu },\ddddot{\mu }\in \text{SAC}\;S_{\mu }}\frac{\left[\;BI(\mu ,v)\right]}{\left[\;BI(\mu ,v)\right]+\sum_{\;\ddddot{\nu }\in \text{SAC}\;S_{\;\ddddot{\nu }}}\left[\;BI(\dddot{\mu },\dddot{\nu })\right]}+\sum_{\bar{\nu },\dddot{\dddot{\mu }}\in \text{SAC}\;S_{v}}\frac{\left[BI(\mu ,v)\right]}{\left[BI(\mu ,v)\right]+\sum_{\dddot{\dddot{\nu }}\in \text{SAC}\;S_{\dddot{\dddot{\nu }}}}\left[BI(\dddot{\dddot{\mu }},\dddot{\dddot{\nu }})\right]}\right)}{N}$$ (5)

- *GI*(*μ*, *ν*): The Global Influence (GI) score of the Association Edge (*μ*, *ν*) that connects SACs *S*_*μ*_ or *S*_*ν*_
- *BI*(*μ*, *ν*): The BI score of the Association Edge (*μ*, *ν*).
- *S*_*μ*_: The SAC containing MKCSS node *μ*.
- $\bar{\mu }$ and $\dddot{\mu }$: Two MKCSS Nodes in SAC *S*_*μ*_. $\dddot{\mu }$ is linked to an MKCSS node $\dddot{\nu }$ that belongs to another SAC *S*_*ν*_
- $BI(\dddot{\mu },\dddot{\nu })$: The BI score of the Association Edge $\left(\dddot{\mu },\dddot{\nu }\right)$.
- *S*_*ν*_: The SAC containing MKCSS node *ν*.
- $\bar{\nu }$ and $\dddot{\dddot{\mu }}$: Two MKCSS nodes in SAC *S*_*ν*_. $\dddot{\dddot{\mu }}$ is linked to an MKCSS node $\dddot{\dddot{\nu }}$ that belongs to another SAC $S_{\ddddot{\ddddot{\nu }}}$
- $BI(\dddot{\dddot{\mu }},\dddot{\dddot{\nu }})$: The BI score of the Association Edge $\left(\dddot{\dddot{\mu }},\dddot{\dddot{\nu }}\right)$.
- *N*: Overall number of nodes that belong to SACs *S*_*μ*_ and *S*_*ν*_

We need to adjust the formula in Eq 5 to keep assigning a higher GI score to the Association Edge (*μ*, *ν*) as the following ratio increases: (1) the number of shortest paths from each node *n* in SACs *S*_*μ*_ and *S*_*ν*_ to the edge (*μ*, *ν*), to (2) the overall number of shortest paths from *n* to the other Association Edges connecting *S*_*μ*_ and *S*_*ν*_ to the remaining SACs in the MRG. The rationale behind this is that the increase in the above ratio is an indicator of the centrality of the Association Edge (*μ*, *ν*) in controlling the flow of information between each of SACs *S*_*μ*_ and *S*_*ν*_ and the remaining SACs in the MRG relative to the centralities of the other Association Edges that can pass the same flow of information. We adjusted the formula in Eq 5 accordingly as shown in Eq 6.


$$GI(\mu ,v)=\frac{\left(\sum_{\bar{\mu },\ddddot{\mu }\in \text{SAC}\;S_{\mu }}\frac{\left[\left|P\;\left(\bar{\mu },\mu \right)\right|\times BI(\mu ,v)\right]}{\left[\left|P\;\left(\bar{\mu },\mu \right)\right|\times BI(\mu ,v)\right]+\sum_{\;\dddot{\nu }\in \text{SAC}\;S_{\;\dddot{\nu }}}\left[\left|P\left(\bar{\mu },\dddot{\mu }\;\right)\right|\times BI(\dddot{\mu },\dddot{\nu })\right]}+\sum_{\bar{\nu },\dddot{\dddot{\mu }}\in \text{SAC}\;S_{v}}\frac{\left[\left|P\left(\bar{\nu },\nu \right)\right|\times BI(\mu ,v)\right]}{\left[\left|P\left(\bar{\nu },\nu \right)\right|\times BI(\mu ,v)\right]+\sum_{\dddot{\dddot{\nu }}\in \text{SAC}\;S_{\ddddot{\ddddot{\nu }}}}\left[\left|P\left(\bar{\nu },\dddot{\dddot{\mu }}\right)\right|\times BI(\dddot{\dddot{\mu }},\dddot{\dddot{\nu }})\right]}\right)}{N}$$ (6)

- $\left|P\left(\bar{\mu },\mu \right)\right|$: Number of shortest paths from MKCSS node $\bar{\mu }$ in SAC *S*_*μ*_ to the Association Edge (*μ*, *ν*).
- $\left|P\left(\bar{\mu },\dddot{\mu }\right)\right|$: Number of shortest paths from MKCSS node $\bar{\mu }$ in SAC *S*_*μ*_ to each other Association Edge $\left(\dddot{\mu },\dddot{\nu }\right)$ connecting SAC *S*_*μ*_ to another SAC $S_{\ddddot{\nu }}$ in the MRG.
- $\left|P\left(\bar{\nu },\nu \;\right)\right|$: Number of shortest paths from MKCSS node $\bar{\nu }$ in SAC *S*_*ν*_ to the Association Edge (*μ*, *ν*).
- $\left|P\left(\bar{\nu },\dddot{\dddot{\mu }}\right)\right|$: Number of shortest paths from MKCSS node $\bar{\nu }$ in SAC *S*_*ν*_ to each other Association Edge $\left(\dddot{\dddot{\mu }},\dddot{\dddot{\nu }}\right)$ connecting SAC *S*_*ν*_ to another SAC $S_{\dddot{\dddot{\nu }}}$ in the MRG.

We now need to consider the impact of nodes’ centralities on the centralities of the Association Edges connecting them. A node’s centrality is manifested by its weight (recall Theorem 1 for how the weight is calculated). Intuitively, the higher the weight of an MKCSS node, the larger is the contribution of the node on the GI score of the Association Edge (*μ*, *ν*), through which the information sent by the node passes. That is, the higher the weights of the nodes sending their information through the Association Edge (*μ*, *ν*), the more influential is the Association Edge. The rationale behind this is that as a node’s weight increases, its influence increases, which in turn reflects in the influence of the Association Edge, through which the information sent by the node passes. That is, as the collective influence of the MKCSS nodes that send their information through the Association Edge (*μ*, *ν*) increase, the influence of the (*μ*, *ν*) in controlling the flow of information throughout the MRG increases at the expense of the other Association Edges. Towards this, we adjusted the formula in Eq 6 as shown in Eq 7.


$$GI(\mu ,v)=\frac{\left(\sum_{\bar{\mu },\ddddot{\mu }\in \text{SAC}\;S_{\mu }}\frac{{\left[\left|P\;\left(\bar{\mu },\mu \right)\right|\times BI(\mu ,v)\right]}^{\;w\left(\bar{\mu }\right)}}{{\left[\left|P\;\left(\bar{\mu },\mu \right)\right|\times BI(\mu ,v)\right]}^{\;w\left(\bar{\mu }\right)}+\sum_{\;\ddddot{\nu }\in \text{SAC}\;S_{\;\ddddot{\nu }}}{\left[\left|P\left(\bar{\mu },\dddot{\mu }\;\right)\right|\times BI(\dddot{\mu },\dddot{\nu })\right]}^{\;w\left(\bar{\mu }\right)}}+\sum_{\bar{\nu },\dddot{\dddot{u}}\in \text{SAC}\;S_{v}}\frac{{\left[\left|P\left(\bar{\nu },\nu \right)\right|\times BI(u,v)\right]}^{\;w\left(\bar{\nu }\right)}}{{\left[\left|P\left(\bar{\nu },\nu \right)\right|\times BI(u,v)\right]}^{\;w\left(\bar{\nu }\right)}+\sum_{\dddot{\dddot{\nu }}\in \text{SAC}\;S_{\ddddot{\ddddot{\nu }}}}{\left[\left|P\left(\bar{\nu },\dddot{\dddot{u}}\right)\right|\times BI(\dddot{\dddot{u}},\dddot{\dddot{\nu }})\right]}^{\;w\left(\bar{\nu }\right)}}\right)}{N}$$ (7)

- $w(\bar{\mu })$: The weight of MKCSS node $\bar{\mu }$.
- $w(\bar{\nu })$: The weight of each other MKCSS node $\bar{\nu }$.

Finally, we aim at using the characteristics of logarithm to capture and further enhance the eigenvector observation/principle [25]. That is, we aim at using logarithm to characterize the "global" (as opposed to "local") influence of an Association Edge in the MRG. By using logarithm, the formula will penalize an Association Edge *exponentially* as the weights of the nodes at its end points decrease and reward it *exponentially* as the weights of these node increase. That is, Association Edges connected to MKCSS nodes with smaller weights are penalized and the ones connected to nodes with larger weights are rewarded exponentially. This helps in accounting for the discrimination between the following two range of variations: (1) the range of variations in large nodes’ weights, and (2) the range of variations in small nodes’ weights. We adjusted Eq 7 accordingly as shown in Eq 8.


$$GI(\mu ,v)=\frac{\left(\sum_{\bar{\mu },\dddot{\mu }\in \text{SAC}\;S_{\mu }}\frac{{\left[\left|P\;\left(\bar{\mu },\mu \right)\right|\times BI(\mu ,v)\right]}^{\;\text{log}{\;}_{2}\;w\left(\bar{\mu }\right)}}{{\left[\left|P\;\left(\bar{\mu },\mu \right)\right|\times BI(\mu ,v)\right]}^{\;{\text{log}}_{2}w\left(\bar{\mu }\right)}+\sum_{\;\ddddot{\nu }\in \text{SAC}\;S_{\;\ddddot{\nu }}}{\left[\left|P\left(\bar{\mu },\dddot{\mu }\;\right)\right|\times BI(\dddot{\mu },\dddot{\nu })\right]}^{\;{\text{log}}_{2}w\left(\bar{\mu }\right)}}+\sum_{\bar{\nu },\dddot{\dddot{\mu }}\in \text{SAC}\;S_{v}}\frac{{\left[\left|P\left(\bar{\nu },\nu \right)\right|\times BI(\mu ,v)\right]}^{\;{\text{log}}_{2}\;w\left(\bar{\nu }\right)}}{{\left[\left|P\left(\bar{\nu },\nu \right)\right|\times BI(\mu ,v)\right]}^{\;{\text{log}}_{2}w\left(\bar{\nu }\right)}+\sum_{\dddot{\dddot{\nu }}\in \text{SAC}\;S_{\ddddot{\ddddot{\nu }}}}{\left[\left|P\left(\bar{\nu },\dddot{\dddot{\mu }}\right)\right|\times BI(\dddot{\dddot{\mu }},\dddot{\dddot{\nu }})\right]}^{{\text{log}}_{2}w\left(\bar{\nu }\right)}}\right)}{N}$$ (8)

**Fig 8:**
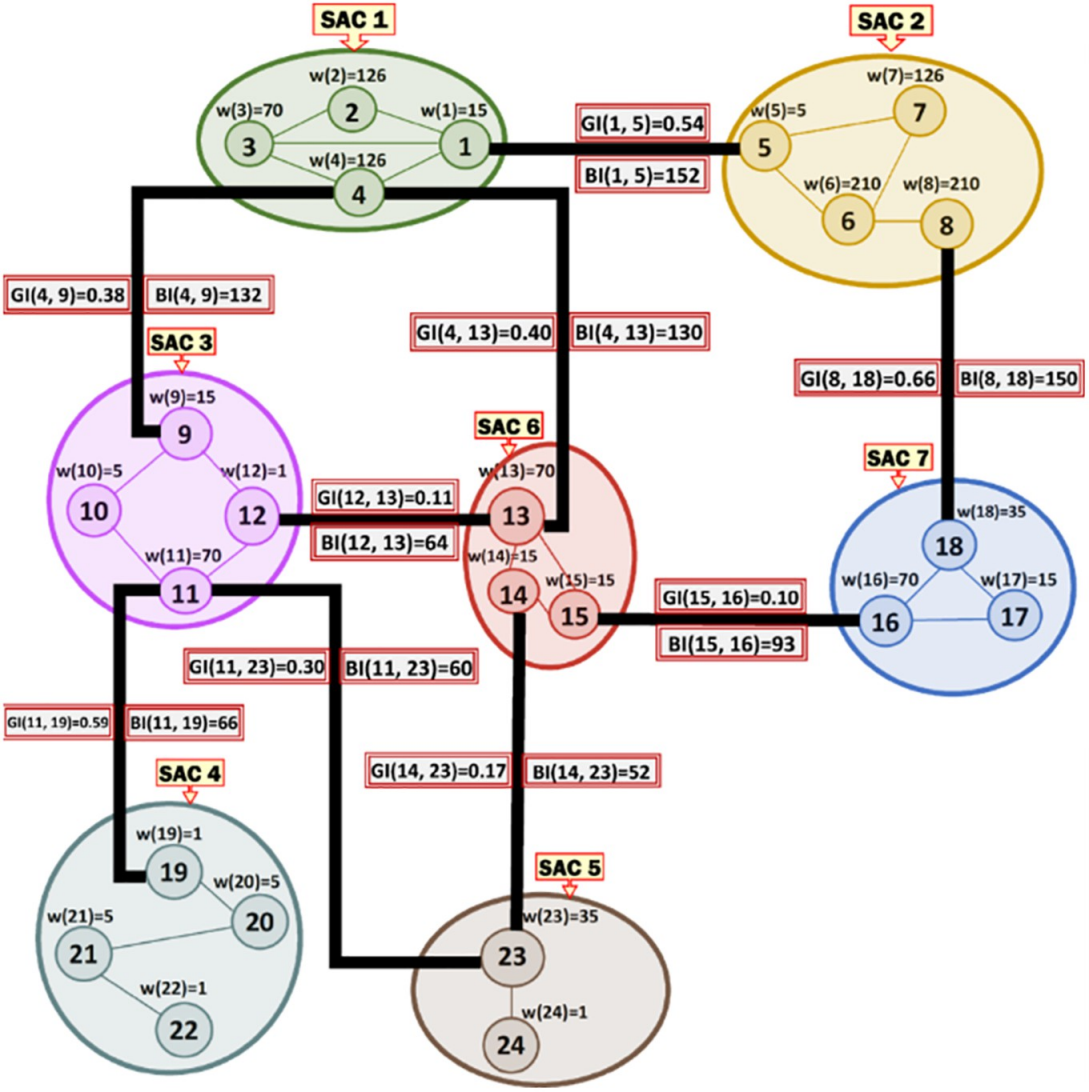
The GI and BI scores of all the Association Edges in our running MRG example after applying Eq 8 (recall Fig 6).

***Running Example 4***: To better illustrate Eq 8‘s composition and components, we show below how the GI score of the Association Edge (1, 5) in our running MRG example is computed using Eq 8 (due to line space restriction, we separated the computations for SACs 1 and 2 and presented the final result in another separate line). Fig 8 shows the GI scores of all the Association Edges in our running MRG example after applying Eq 8.


$$\text{SAC}\;1=\frac{\overset{\text{\#}\;\text{paths}\times \text{BI}(1,5)}{\overset{︷}{{\left(1\times 152\right)}^{{\text{log}}_{2}15\overset{\text{w}(1)}{\leftarrow }}}}}{\underset{\text{\#}\;\text{paths}\times \text{BI}(1,5)}{\underset{︸}{{\left(1\times 152\right)}^{{\text{log}}_{2}15}}}+\underset{\text{\#}\;\text{paths}\times \text{BI}(4,\;9)}{\underset{︸}{{\left(1\times 132\right)}^{{\text{log}}_{2}15}}}+\underset{\text{\#}\;\text{paths}\times \text{BI}(4,\;13)}{\underset{︸}{{\left(1\times 130\right)}^{{\text{log}}_{2}15}}}}+\frac{\overset{\text{\#}\;\text{paths}\times \text{BI}(1,5)}{\overset{︷}{{\left(1\times 152\right)}^{{\text{log}}_{2}126\overset{\text{w}(2)}{\leftarrow }}}}}{\underset{\text{\#}\;\text{paths}\times \text{BI}(1,5)}{\underset{︸}{{\left(1\times 152\right)}^{{\text{log}}_{2}126}}}+\underset{\text{\#}\;\text{paths}\times \text{BI}(4,\;9)}{\underset{︸}{{\left(2\times 132\right)}^{{\text{log}}_{2}126}}}+\underset{\text{\#}\;\text{paths}\times \text{BI}(4,\;13)}{\underset{︸}{{\left(2\times 130\right)}^{{\text{log}}_{2}126}}}}+\frac{\overset{\text{\#}\;\text{paths}\times \text{BI}(1,5)}{\overset{︷}{{\left(2\times 152\right)}^{{\text{log}}_{2}70\overset{\text{w}(3)}{\leftarrow }}}}}{\underset{\text{\#}\;\text{paths}\times \text{BI}(1,5)}{\underset{︸}{{\left(2\times 152\right)}^{{\text{log}}_{2}70}}}+\underset{\text{\#}\;\text{paths}\times \text{BI}(4,\;9)}{\underset{︸}{{\left(1\times 132\right)}^{{\text{log}}_{2}70}}}+\underset{\text{\#}\;\text{paths}\times \text{BI}\;(4,13)}{\underset{︸}{{\left(1\times 130\right)}^{{\text{log}}_{2}70}}}}+\frac{\overset{\text{\#}\;\text{paths}\times \text{BI}\;(1,5)}{\overset{︷}{{\left(1\times 152\right)}^{{\text{log}}_{2}126\overset{\text{w}(4)}{\leftarrow }}}}}{\underset{\text{\#}\;\text{paths}\times \text{BI}(1,5)}{\underset{︸}{{\left(1\times 152\right)}^{{\text{log}}_{2}126}}}+\underset{\text{\#}\;\text{paths}\times \text{BI}(4,\;9)}{\underset{︸}{{\left(1\times 132\right)}^{{\text{log}}_{2}126}}}+\underset{\text{\#}\;\text{paths}\times \text{BI}(4,\;13)}{\underset{︸}{{\left(1\times 130\right)}^{{\text{log}}_{2}126}}}}=2.22$$

$$\text{SAC}\;2=\frac{\overset{\text{\#}\;\text{paths}\times \text{BI}(1,5)}{\overset{︷}{{\left(1\times 152\right)}^{{\text{log}}_{2}5\overset{\text{w}(5)}{\leftarrow }}}}}{\underset{\text{\#}\;\text{paths}\times \text{BI}(1,5)}{\underset{︸}{{\left(1\times 152\right)}^{{\text{log}}_{2}5}}}+\underset{\text{\#}\;\text{paths}\times \text{BI}(8,\;18)}{\underset{︸}{{\left(1\times 150\right)}^{{\text{log}}_{2}5}}}\;}+\frac{\overset{\text{\#}\;\text{paths}\times \text{BI}(4,5)}{\overset{︷}{{\left(1\times 152\right)}^{{\text{log}}_{2}210\overset{\text{w}(6)}{\leftarrow }}}}}{\underset{\text{\#}\;\text{paths}\times \text{BI}\;(4,5)}{\underset{︸}{{\left(1\times 152\right)}^{{\text{log}}_{2}210}}}+\underset{\text{\#}\;\text{paths}\times \text{BI}\;(4,5)}{\underset{︸}{{\left(1\times 150\right)}^{{\text{log}}_{2}210}}}\;}+\frac{\overset{\text{\#}\;\text{paths}\times \text{BI}(1,5)}{\overset{︷}{{\left(1\times 152\right)}^{{\text{log}}_{2}126\overset{\text{w}(7)}{\leftarrow }}}}}{\underset{\text{\#}\;\text{paths}\times \text{BI}(1,5)}{\underset{︸}{{\left(1\times 152\right)}^{{\text{log}}_{2}126}}}+\underset{\text{\#}\;\text{paths}\times \text{BI}(8,\;18)}{\underset{︸}{{\left(1\times 150\right)}^{{\text{log}}_{2}126}}}\;}+\frac{\overset{\text{\#}\;\text{paths}\times \text{BI}(1,5)}{\overset{︷}{{\left(1\times 152\right)}^{{\text{log}}_{2}210\overset{\text{w}(8)}{\leftarrow }}}}}{\underset{\text{\#}\;\text{paths}\times \text{BI}(1,5)}{\underset{︸}{{\left(1\times 152\right)}^{{\text{log}}_{2}210}}}+\underset{\text{\#}\;\text{paths}\times \text{BI}(8,\;18)}{\underset{︸}{{\left(1\times 150\right)}^{{\text{log}}_{2}210}}}\;}=2.08$$

$$\text{GI}(1,5)=\frac{2.22+2.08}{8}=0.54$$

### Uncovering missing Association Edges in the MKCSS Relationship Tree

Most often, some links in social networks are not revealed as a result of members who did not fully disclose and declare their memberships to all communities. It is imperative for uncovering such missing Association Edges prior to detecting cross-communities because they may contain valuable cross-membership information. First, ID_CC converts the MRG into a tree data structure for the ease of uncovering missing Association Edges connecting hierarchical interrelated SACs. A tree is a connected acyclic graph that reflects the hierarchical tree structure of the graph. The root of the tree is a parent node and is not a child of any node. Each node has only one parent and may have ancestor nodes. A leaf node is a node that does not have a child node. We call the resulting converted tree data structure the MKCSS Relationship Tree.

We followed the following procedure for converting a MRG into a MKCSS Relationship Tree. Let *ɳ* and *ɱ* be two adjacent neighbouring nodes in the MRG. If *ɱ* is closer to the root node than *ɳ*, we consider *ɱ* to be the parent of *ɳ* in the MKCSS Relationship Tree. Thus, we construct the MKCSS Relation Tree by identifying the parent of each node *ɳ* as follows. From the set of Association Edges connected to *ɳ*, we select the Association Edge *Ҿ* with the highest GI score. If *ɳ* and *ɱ* are the end points of *Ҿ*, we consider *ɱ* to be the parent of *ɳ*. The rationale behind this is that, from among the set of nodes connected to *ɳ*, *ɱ* is the most closely associated with *ɳ*.

**Fig 9:**
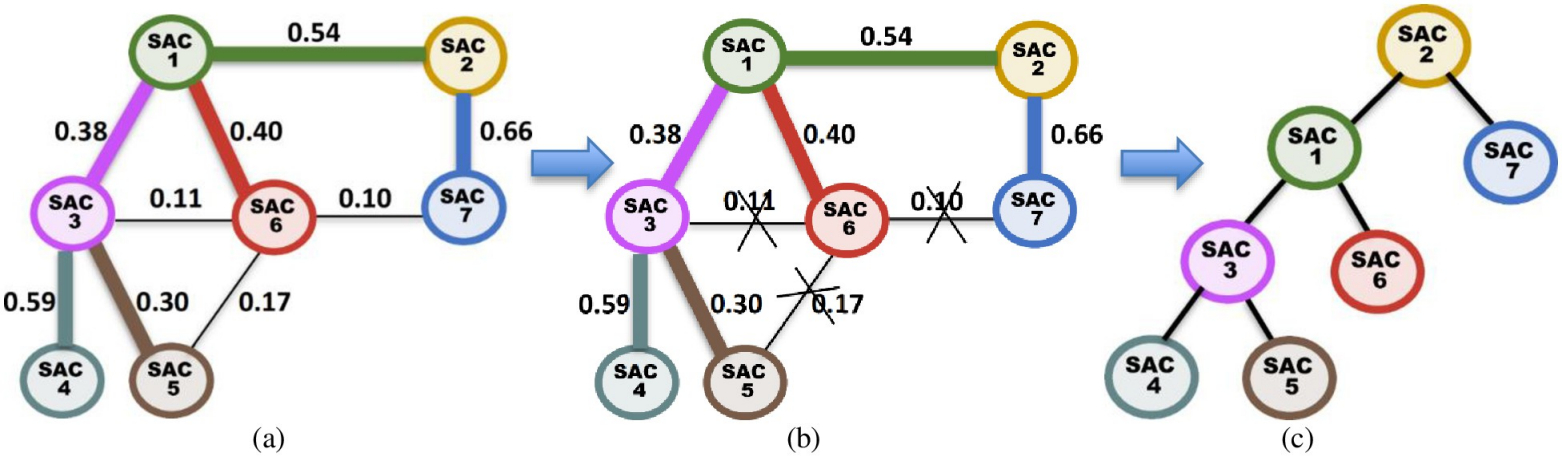
(a) The Association Edge with the highest GI score connected to each SAC node in the MRG (for easy reference, each node and the Association Edge with the highest GI score connected to it are marked with the same colour), (b) each Association Edge *Ҿ* that will be removed from the MRG because the two nodes at its end points are connected to other Association Edge, whose GI scores are higher than that of *Ҿ*, and (c) the resulting MKCSS Relationship Tree.

***Running Example 5***: Consider our running MRG example shown in Fig 8. Fig 9-a shows the Association Edge with the highest GI score connected to each SAC node in the MRG. Fig 9-b shows each Association Edge *Ҿ* that will be removed from the MRG because the two nodes at its end points are connected to other Association Edge, whose GI scores are higher than that of *Ҿ*. Fig 9-c shows the resulting MKCSS Relationship Tree.

### Uncovering implicit Association Edges using the concept of RLCA

**Fig 10:**
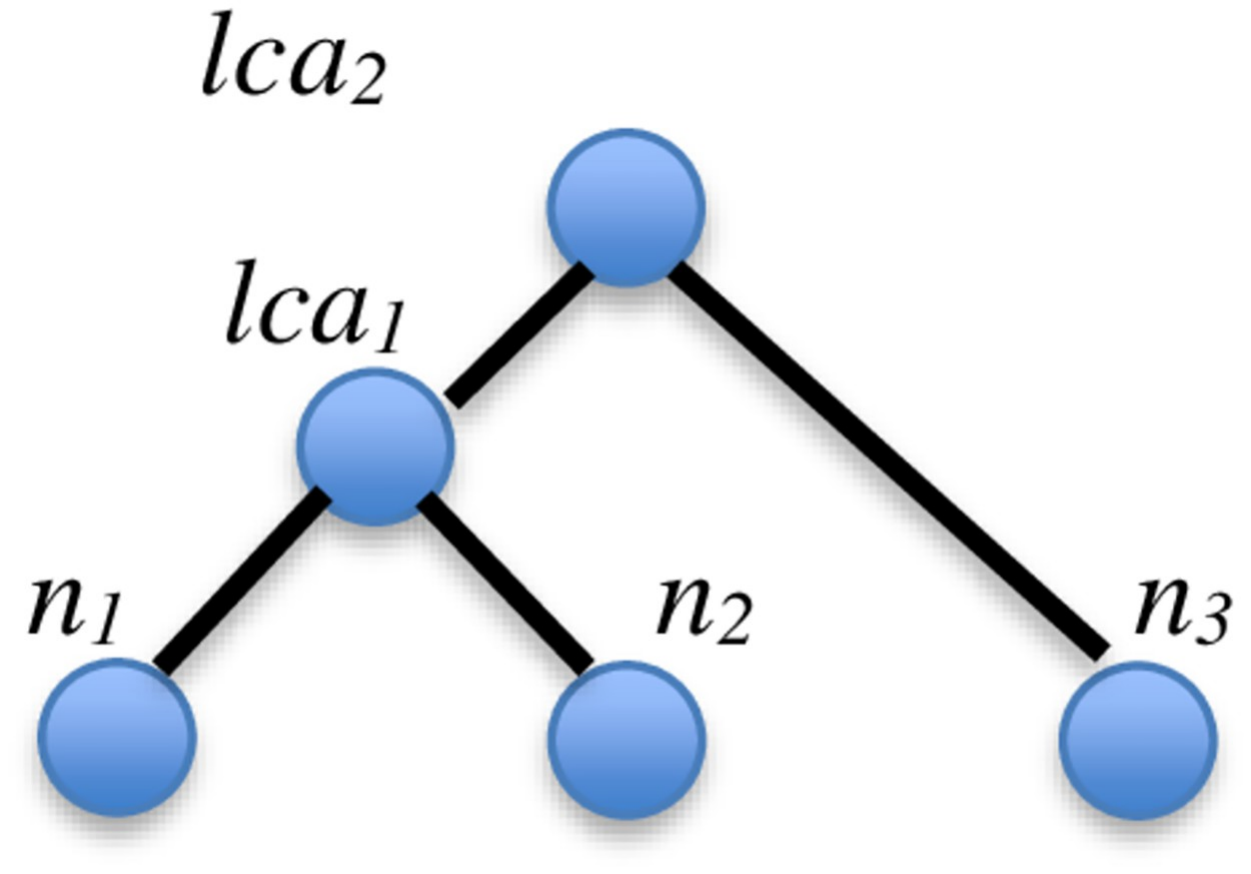
Illustration of the RLCA concept.

We propose the concept of Relevant Lowest Common Ancestor (RLCA) for uncovering missing (i.e., implicit) Association Edges. As plotted in Fig 10, let the Lowest Common Ancestor (LCA) of two nodes *n*_*1*_ and *n*_*2*_ be a node *lca*_*1*_. If: (1) some node *lca*_*2*_ is an ancestor of node *lca*_*1*_, (2) *lca*_*2*_ is the LCA of nodes *n*_*1*_ and *n*_*3*_, and (3) nodes *n*_*3*_ and *n*_*2*_ share some social characteristics, we can infer that node *n*_*3*_ is related to nodes *n*_*1*_. We call *lca*_*2*_ the RLCA of nodes *n*_*3*_ and *n*_*1*_.

**Fig 11:**
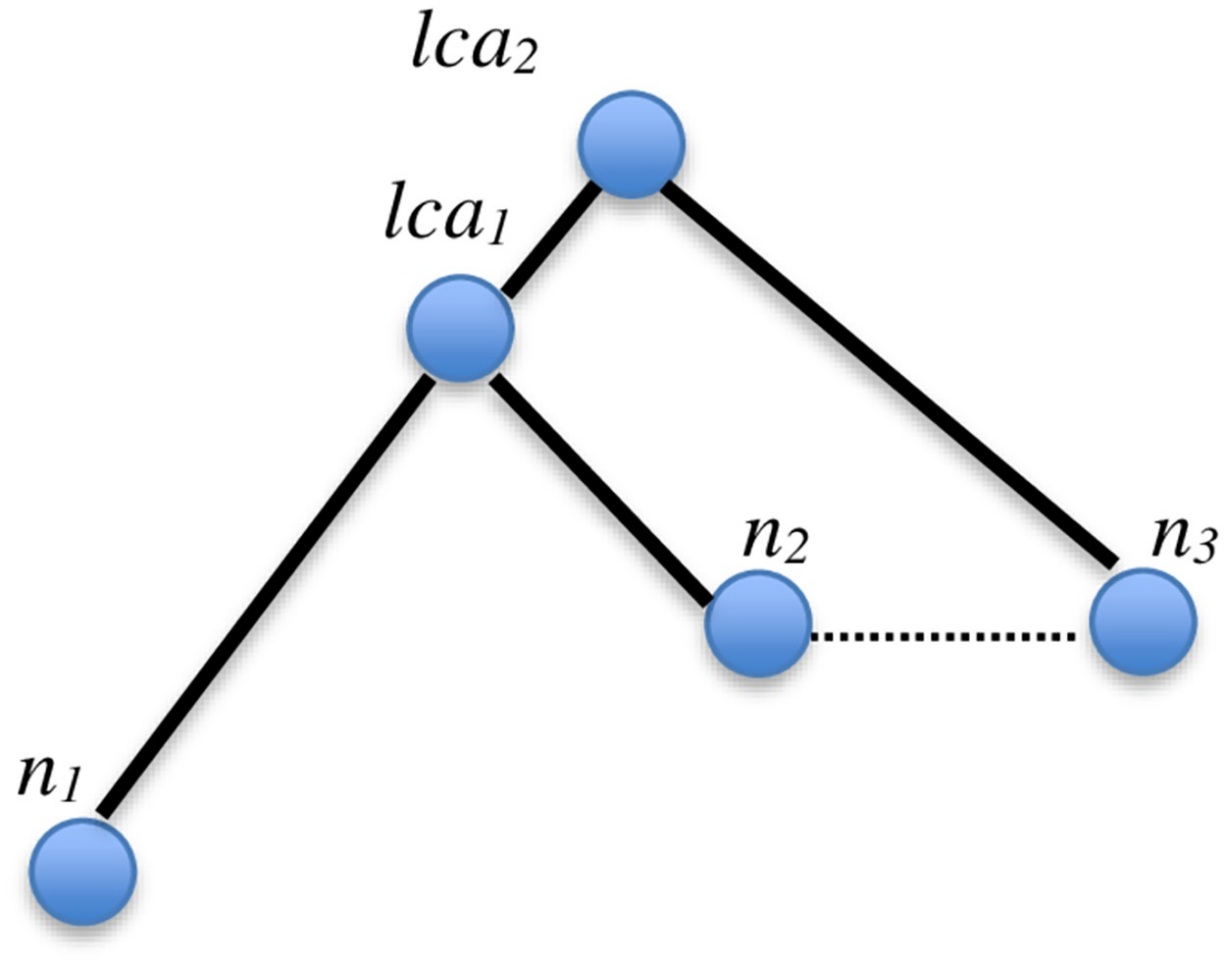
Since the subtree conforms to the RLCA concept, we can deduce an implicit Association Edge connecting nodes *n*_*3*_ and *n*_*1*_, if nodes *n*_*2*_ and *n*_*3*_ are connected by an edge in the MRG.

By applying the concept of RLCA, we can uncover implicit Association Edges in the MRG as follows. If the characteristics shared between nodes *n*_*2*_ and *n*_*3*_ in Fig 10 are manifested by an Association Edge in the MRG, we can deduce an implicit Association Edge connecting nodes *n*_*3*_ and *n*_*1*_ as depicted in Fig 11. The above procedure is applied successively on the MKCSS Relationship Tree to identify all subtrees that satisfy the RLCA concept. An implicit Association Edge is uncovered for each conforming subtree.

**Fig 12:**
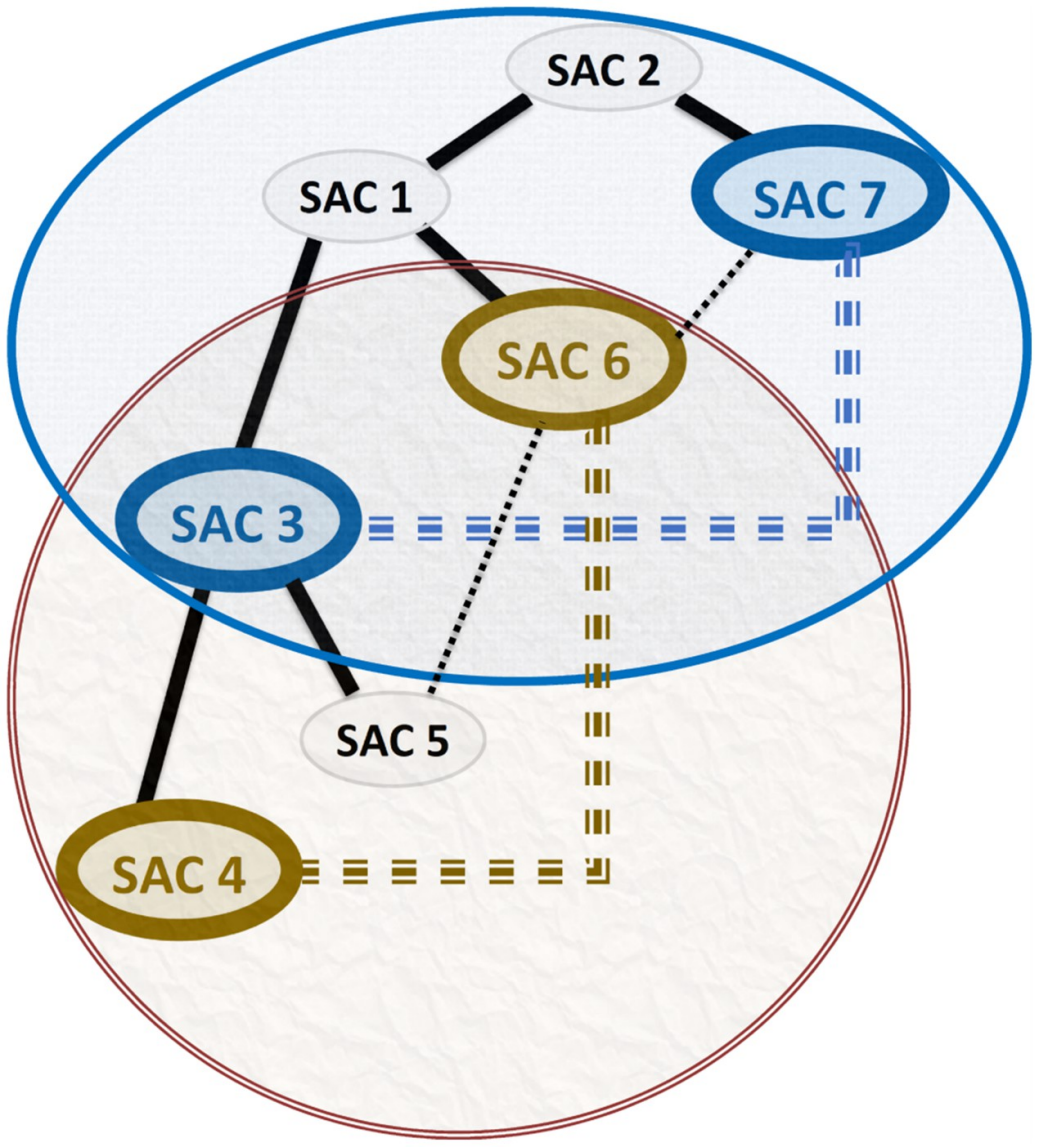
Two subtrees conforming to the RLCA concept. In the first subtree, since SACs 7 and 6 are connected by an edge, SACs 7 and 3 are linked by an implicit Association Edge. In the second subtree, since SACs 6 and 5 are connected by an edge, SACs 6 and 4 are linked by an implicit Association Edge.

***Running Example 6***: By applying the RLCA concept against the MKCSS Relationship Tree of our running example shown in Fig 9-a, we will discover two subtrees conforming to the RLCA concept as shown in Fig 12. In the first subtree, since SACs 7 and 6 are connected by an edge (recall the MRG in Fig 3), SACs 7 and 3 are linked by an implicit Association Edge. In the second subtree, since SACs 6 and 5 are connected by an edge (recall the MRG in Fig 3), SACs 6 and 4 are linked by an implicit Association Edge.

### Efficiently uncovering implicit Association Edges

**Fig 13:**
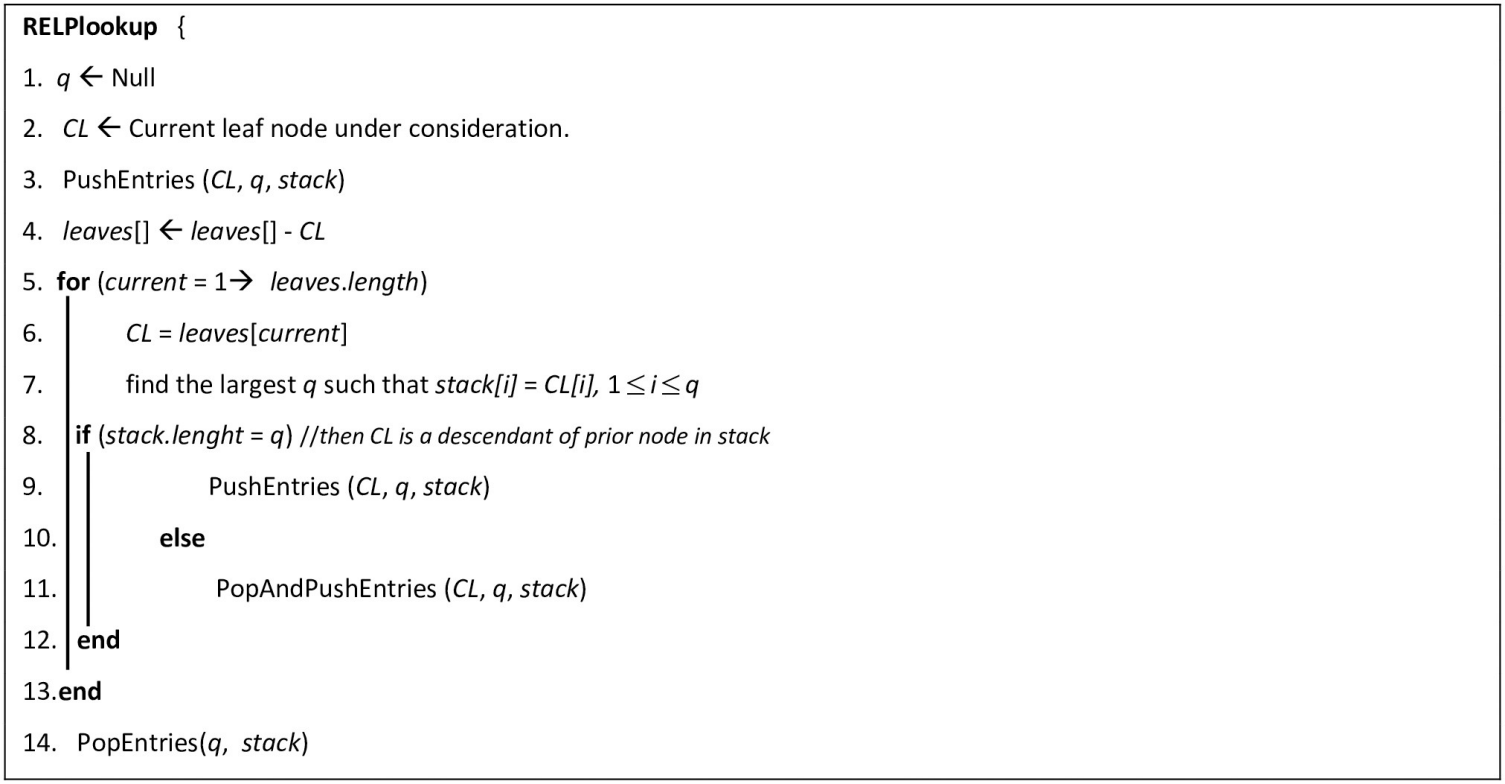
Algorithm RELPlookup.

We constructed an algorithm called RELPlookup (Fig 13) to efficiently uncover implicit edges. The algorithm applies the RLCA concept on MKCSS Relationship Trees using a stack-based sort-merge approach. The algorithm employs a stack, with the head of each stack node being a descendant of the stack node below it. The idea is to perform one single-merge pass over the nodes and conceptually merge them into rooted trees containing the lead nodes. First, the nodes of the MKCSS Relationship Tree are labelled with Dewey IDs. We formalize the Dewey ID concept in Definition 5.

**Fig 14:**
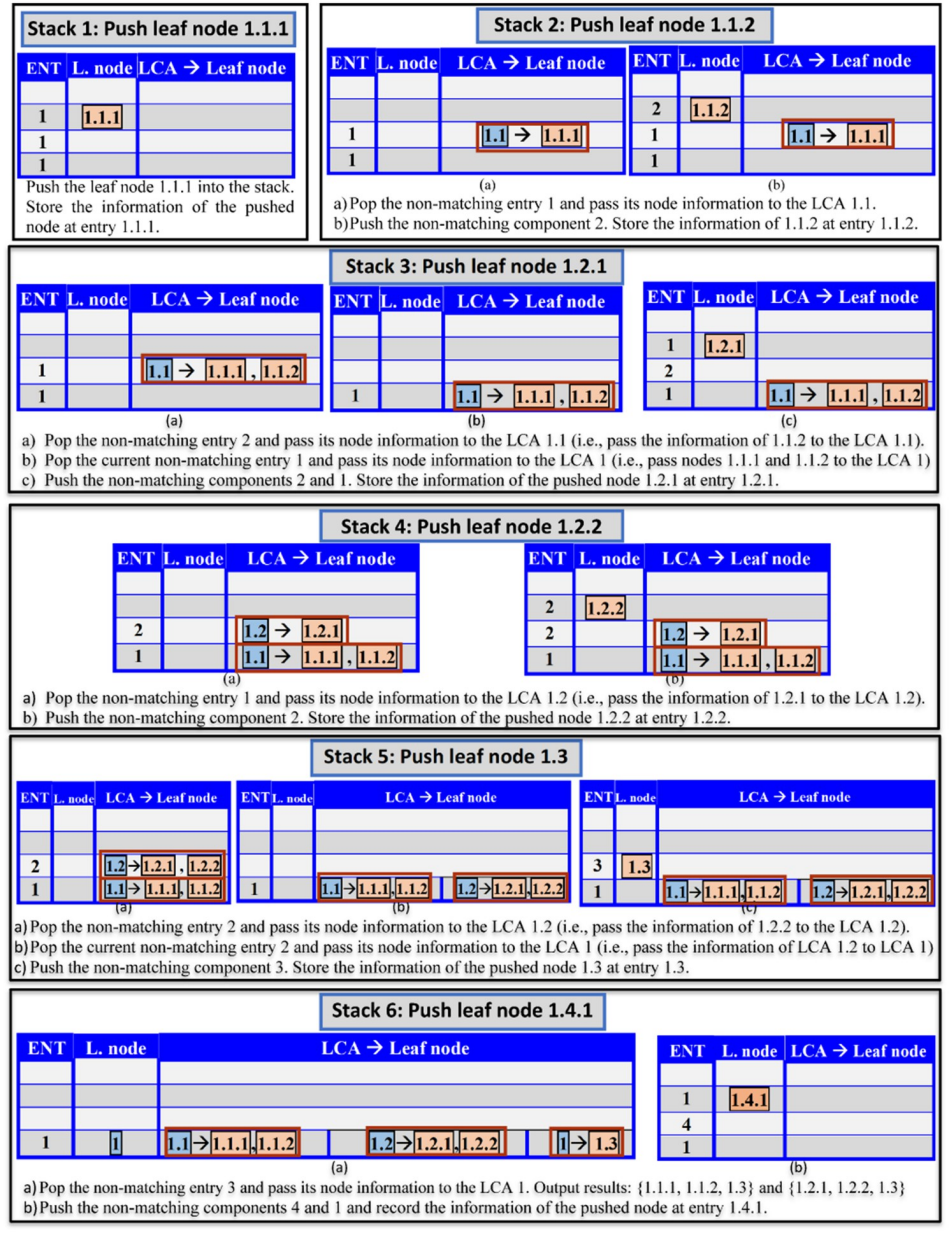
(a) The MKCSS Relationship Tree described in Example 7 labelled with Dewey IDs, and (b) the states of the stack produced by applying algorithm RELPlookup (Fig 13) on the tree in (a). ENT stands for “Entry” and L. node stands for “Leaf node”.

*Definition 5—Dewey ID*: A Dewey ID of a node *n*_*1*_ is a sequence of components. Each component is a sequence of digits separated by decimal points. Each component represents the Dewey ID of an ancestor node *n*_*2*_ of *n*_*1*_. The component to the left of the last decimal point of the Dewey ID of *n*_*1*_ is the parent node of *n*_*1*_. Dewey IDs are assigned to nodes based on the Depth First Search. When the sequence of components in the Dewey ID of *n*_*1*_ are read from left to right, they reveal the chain of ancestors of *n*_*1*_, starting from the root node. For example, consider the Dewey ID of SAC 1.1.1 in Fig 14. It reveals that the Dewey ID of the root SAC is 1. It also reveals that the Dewey ID of the parent of SAC 1.1.1 is 1.1.

**Fig 15:**
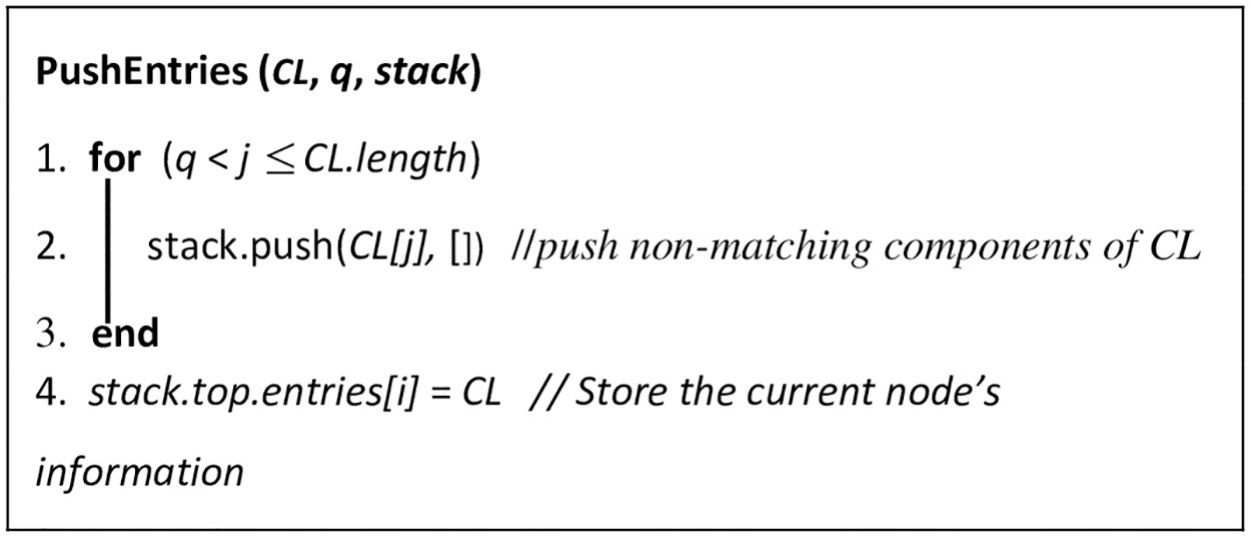
Subroutine PushEntries.

**Fig 16:**
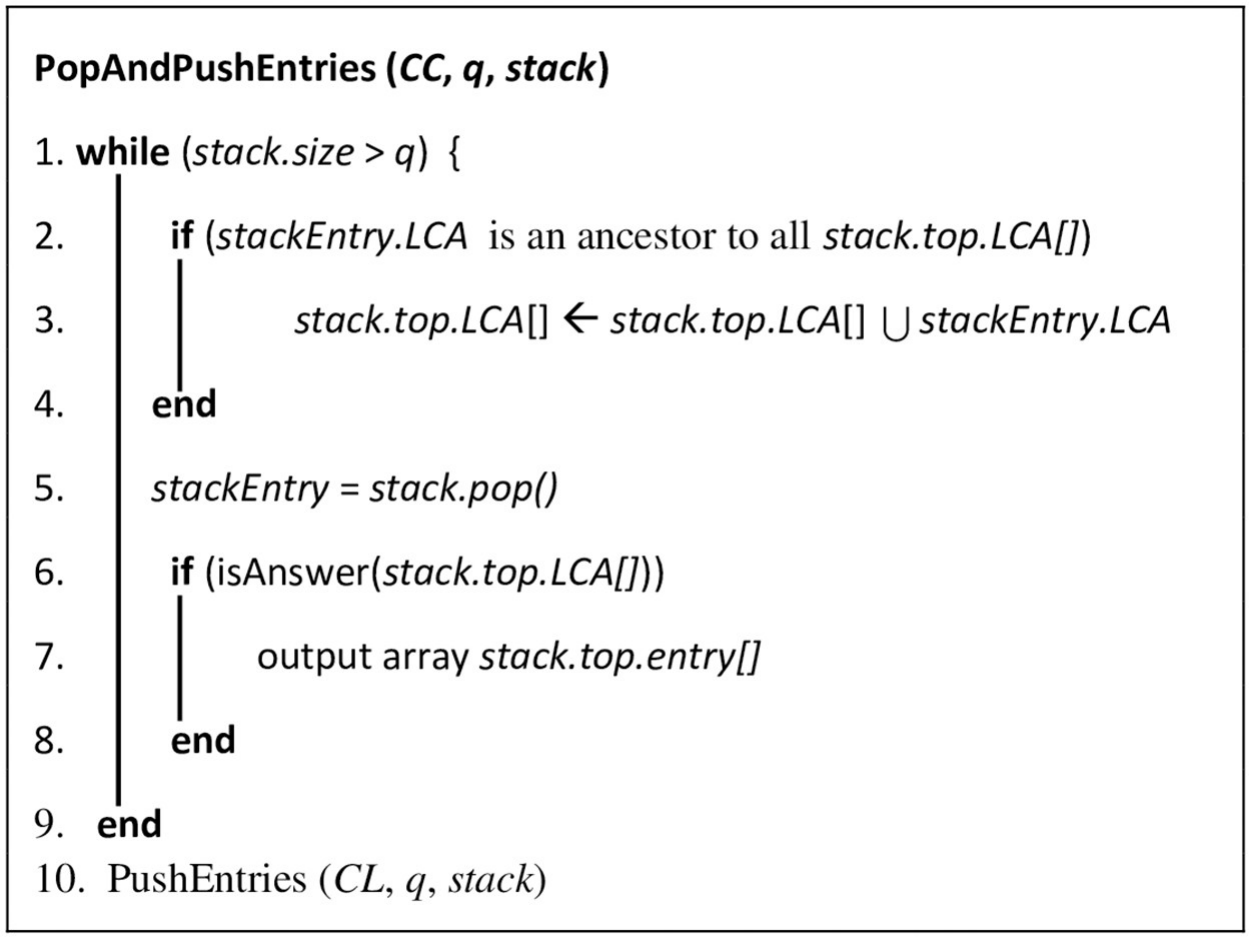
Subroutine PopAndPushEntries.

**Fig 17:**
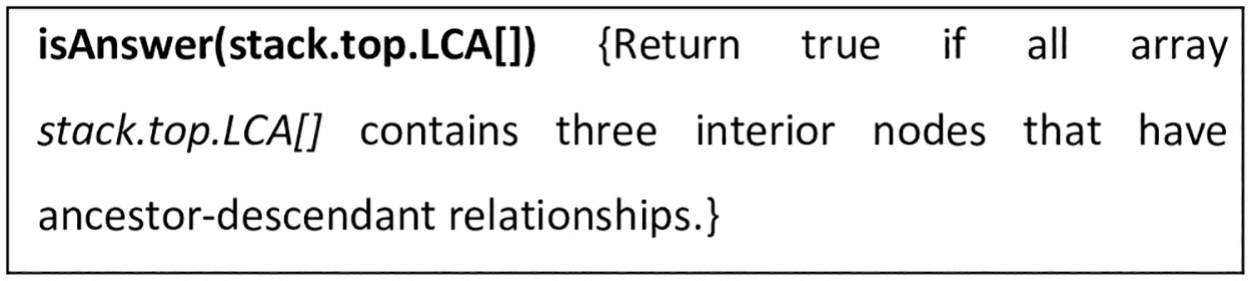
Subroutine isAnswer.

The input to the algorithm is an array called *leaves[]*, which contains the Dewey IDs of the leaf nodes in the tree. Each iteration of the algorithm produces a new stack state. Each stack entry has three array components (*ENT*, *Leaf node*, and *LCA-Leaf node*), where: ENT is the entry of the node pushed into the stack, Leaf node is the Dewey ID of the leaf node pushed into the stack, and *LCA-Leaf node* is the pair of LCA and the leaf nodes passed to this LCA from entries popped out of the stack. If *d*_*i*_, *d*_*j*_,…, and *d*_*k*_ are the Dewey ID components in the stack from the bottom entry to the stack entry, then (1) the stack entry represents the SAC, whose Dewey ID is *d*_*i*_, *d*_*j*_,…, and *d*_*k*_, and (2) the bottom entry represents the root SAC node, whose Dewey ID is *d*_*i*_. The symbol *q* in line 7 of Algorithm RELPlookup represents the number of Dewey ID components of a pushed node that match the components in the entry of the current stack state. If *q* equals the number of Dewey components in the current stack state, the currently processed leaf node is a descendant of the priorly processed node. If this is the case, subroutine PushEntries (Fig 15) is called to push into the stack the non-matching Dewey components of the current node. Otherwise, subroutine PopAndPushEntries (Fig 16) is called to perform the following: (1) pop the non-matching Dewey components of the current node, and (2) push the non-matching Dewey components. Subroutine isAnswer (Fig 17) outputs the results, if: (1) array *LCA-Leaf node* contains information passed from at least two LCAs, (2) one of these LCAs is an ancestor of the other LCA, and (3) the ancestor and descendant LCAs contains the information of three leaf nodes. The three leaf nodes will be output as the result.

**Fig 18:**
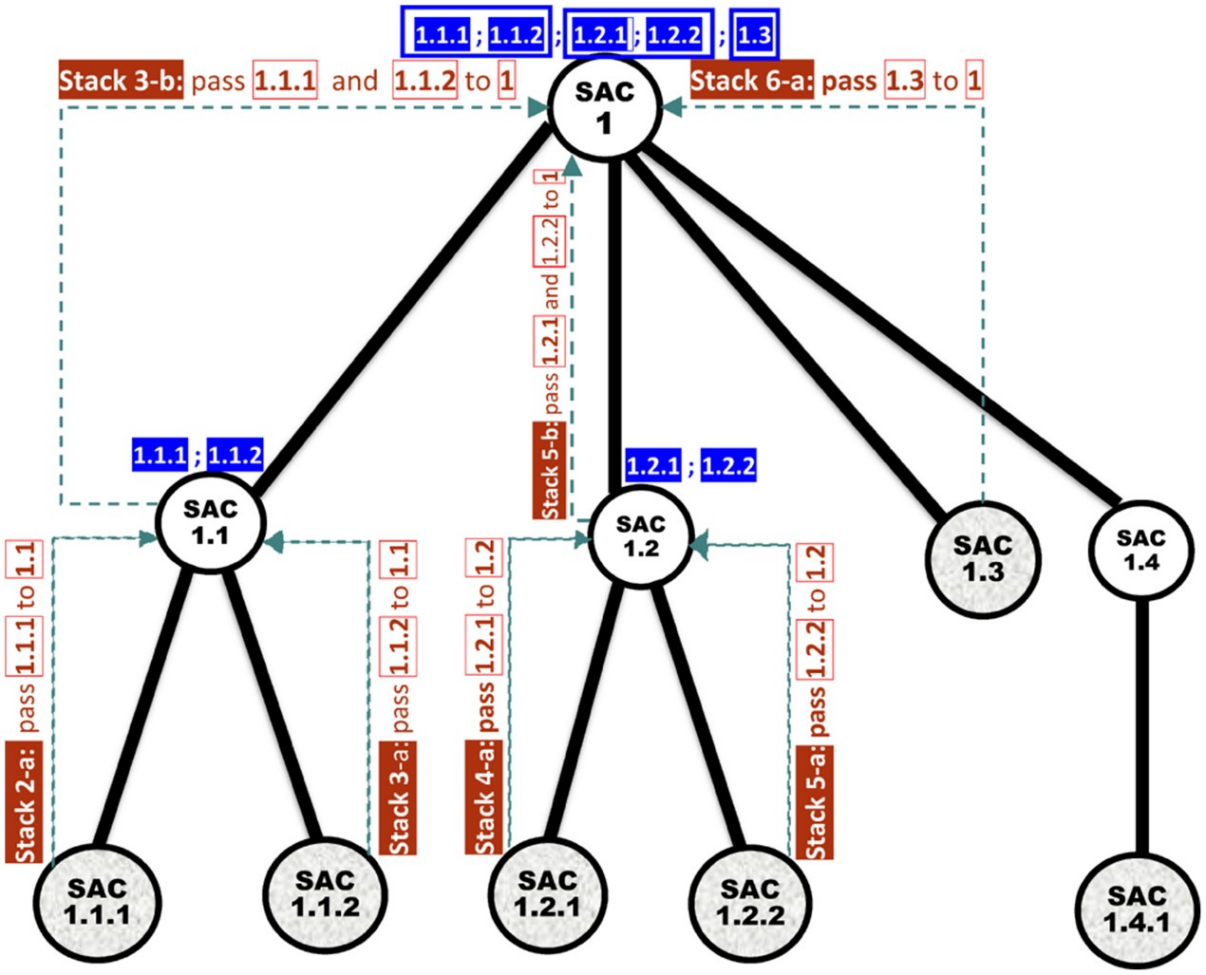
The MKCSS Relationship Tree in Fig 14-a after being annotated with the processing steps of Algorithm RELPlookup (Fig 13) that created the states of the stacks in Fig 14-b. The arrows and texts marked in brown colour describe the processing steps that created the stack states. The sets of nodes on top of SAC 1 are the two results produced by the algorithm: {1.1.1, 1.1.2, 1.3} and {1.2.1, 1.2.2, 1.3}.

***Running Example 7***: Let us apply algorithm RELPlookup (Fig 13) and its subroutines on the MKCSS Relationship Tree shown in Fig 14-a. First, the Dewey IDs of the leaf nodes will be stored in the array *leaves*: *leaves[]* = [1.1.1, 1.1.2, 1.2.1, 1.2.2, 1.3, 1.4.1]. The stack is initially empty. Fig 14-b shows the stack states produced by the algorithm. In Fig 18, the MKCSS Relationship Tree is annotated with the algorithm’s processing steps that created the states of the stacks in Fig 14-b. As Figs 14-b and 18 show, there are two results: {1.1.1, 1.1.2, 1.3} and {1.2.1, 1.2.2, 1.3}. If node 1.3 is connected by an edge in the MRG with one of the nodes in each of the two sets, node 1.3 and the other node in the set are connected by an implicit Association Edge. For example, if nodes 1.3 and 1.1.1 in the first set are connected by an edge in the MRG, then nodes 1.3 and 1.1.2 are connected by an implicit Association Edge.

### Computing the Global Influences of the implicit Association Edges

**Fig 19:**
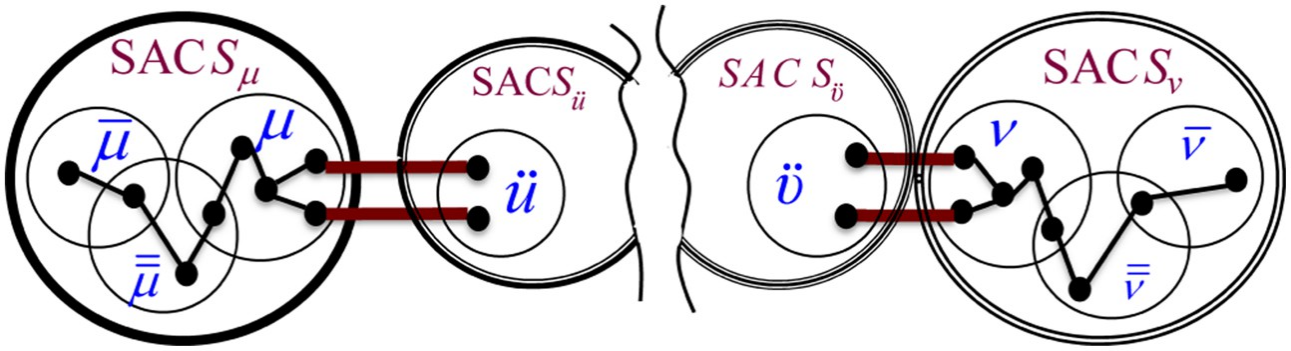
Generic notations used for constructing the formula in Eq 9 for computing the BI of an implicit Association Edge (*μ*, *ν*).

To compute the GIs of the uncovered implicit Association Edges, we need to first compute their BIs. We need to adjust the BI formula in Eq 4 to accommodate the characteristics that are specific to implicit Association Edges. Since not all implicit Association Edges link neighbouring SACs, the shared nodes between these SACs may have different number of cross-members. For example, consider an implicit Association Edge connecting MKCSSs 14 and 19 (recall Fig 2). MKCSS 19 has only one cross-member and MKCSS 14 has four cross-members. However, Eq 4 considers only equal number of cross-members between two neighbouring SACs. Therefore, we need to adjust Eq 4 accordingly. Based on the above observation, we constructed the formula in Eq 9 for computing the BI score of implicit Association Edges using the generic notations in Fig 19. The formula is constructed based on generic un-neighbouring SACs *S*_*u*_ and *S*_*ν*_ connected by an implicit Association Edge (*μ*, *ν*) as shown in Fig 19. The edge connects the two SACs through the cross-members of MKCSSs *u* and *ν*. After computing the BIs, the GIs of these implicit edges will be computed using the formula presented in Eq 8.


$$BI(\mu ,\nu )=\left|\;\mu \cap ü\right|\times \left(\frac{\sum_{\bar{\mu },\bar{\bar{\mu }}\in \text{SAC}\;S_{\mu },ü\in \text{SAC}\;S_{ü}}\left(\sigma (\bar{\mu },\mu )\times \left(\left|\bar{\mu }\right|-\left|\bar{\mu }\cap \bar{\bar{\mu }}\right|\right)\times \left|\bar{\mu }\cap \bar{\bar{\mu }}\right|\right)\;}{2{\;}^{{\text{log}}_{2}\left|\;\mu \cap ü\right|}}\right)+\left|\;\nu \cap ϋ\right|\times \left(\frac{\sum_{\bar{\nu },\bar{\bar{\nu }}\in \text{SAC}\;S_{\nu },ϋ\in \text{SAC}\;S_{ϋ}}\left(\sigma (\bar{\nu },\nu )\times \left(\left|\bar{\nu }\right|-\left|\bar{\nu }\cap \bar{\bar{\nu }}\right|\right)\times \left|\bar{\nu }\cap \bar{\bar{\nu }}\right|\right)}{2{\;}^{{\text{log}}_{2}\left|\;\nu \cap ϋ\right|}}\right)$$ (9)

**Fig 20:**
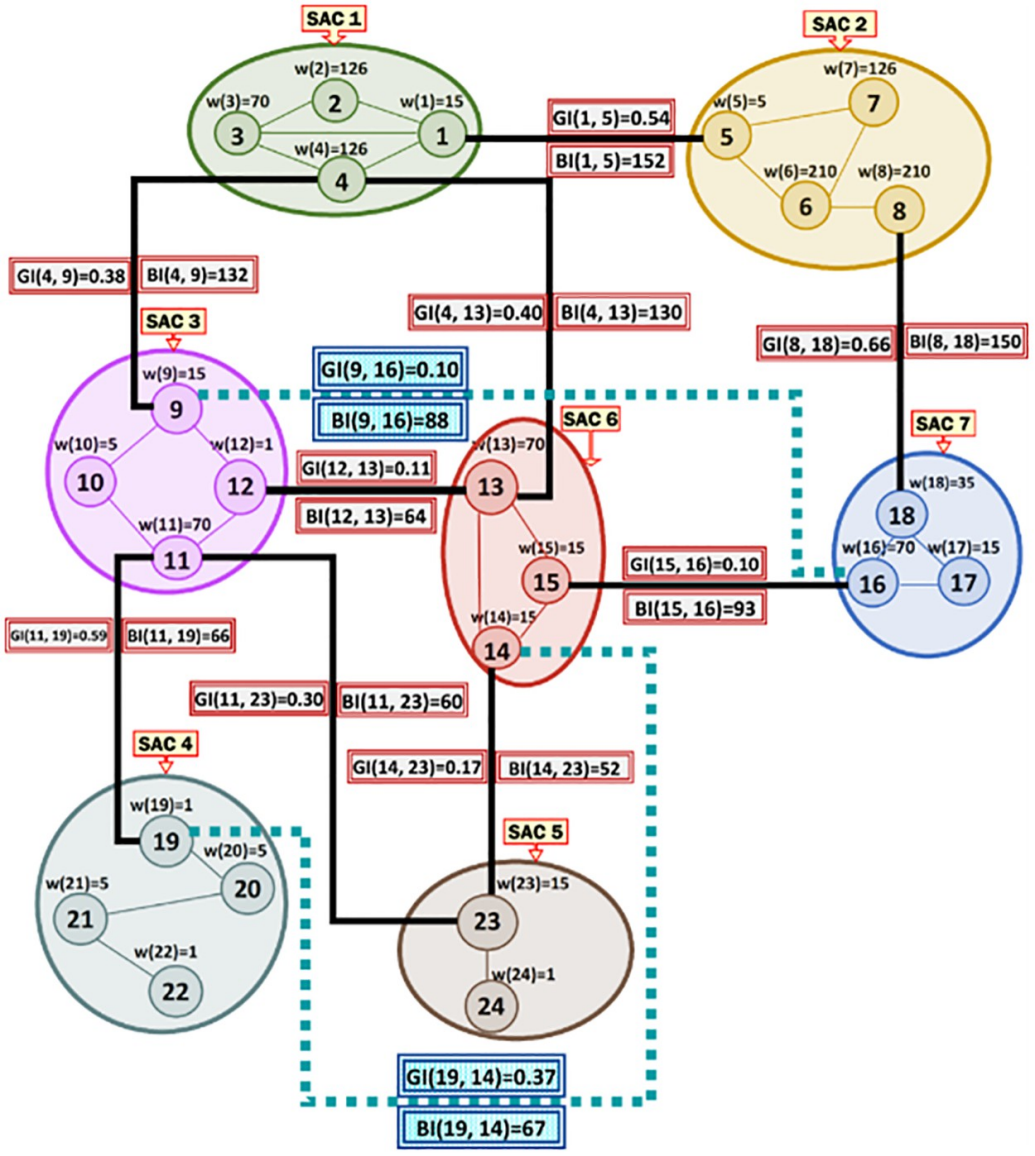
The BIs and GIs of all Association Edges in our running MRG example including the BIs and GIs of the implicit Association Edges connecting SACs 7 and 3 and SACs 6 and 4.

***Running Example 8***: As demonstrated in Fig 12, there are two implicit Association Edges in our running MRG example connecting SACs 7 and 3 and SACs 6 and 4. Fig 20 shows the BI scores of these implicit Association Edges after applying Eq 9. Fig 20 shows both the BIs and GIs of all Association Edges in our running MRG example including the implicit Association Edges.

## Uncovering the densest multi-profiled cross-sacs to which an active user belongs

In this section, we describe the methodology adopted by ID_CC for inferring the *densest* multi-profiled cross-SACs, to which an active user belongs. The larger the number of implicitly detected SACs, to which the active user belongs, the denser and more specific is the multi-profiled cross-community identified by the system for the user. ID_CC assigns the active user to the densest and most granular multi-profiled cross-SACs that matches his/her own social traits. Towards this, it employs a mechanism that can implicitly infer the SACs, to which the user belongs but were not revealed by the user. The mechanism adopted by ID_CC is based on applying the Maximum Spanning Tree (MaxST) algorithm [22, 23] on the revised MKCSS Relationship Tree (i.e., the one containing *all* Association Edges including the implicitly identified ones). All SAC nodes located in the path of the MaxST that connects the user’s revealed (i.e., already known) SAC nodes, will be considered the comprehensive list of SACs, to which the user belongs. The extra SACs in the list (excluding the ones declared by the user) are *implicitly* identified. The resulting intersection of all SACs in the list is the densest and most granular multi-profiled cross-SACs that matches the user’s own social traits. This technique considers all cross-profiles that come to existence from the interrelations between overlapped social profiles. We construct the MaxST based on the GI scores of the Association Edges. A MaxST is a tree that spans all the SAC nodes in the MKCSS Relationship Tree. The sum of the GI scores of the Association Edges connecting the nodes is the largest among all other trees that span all the nodes. The MaxST can be computed using Kruskal’s algorithm [22] after multiplying the GIs values by -1.

**Fig 21:**
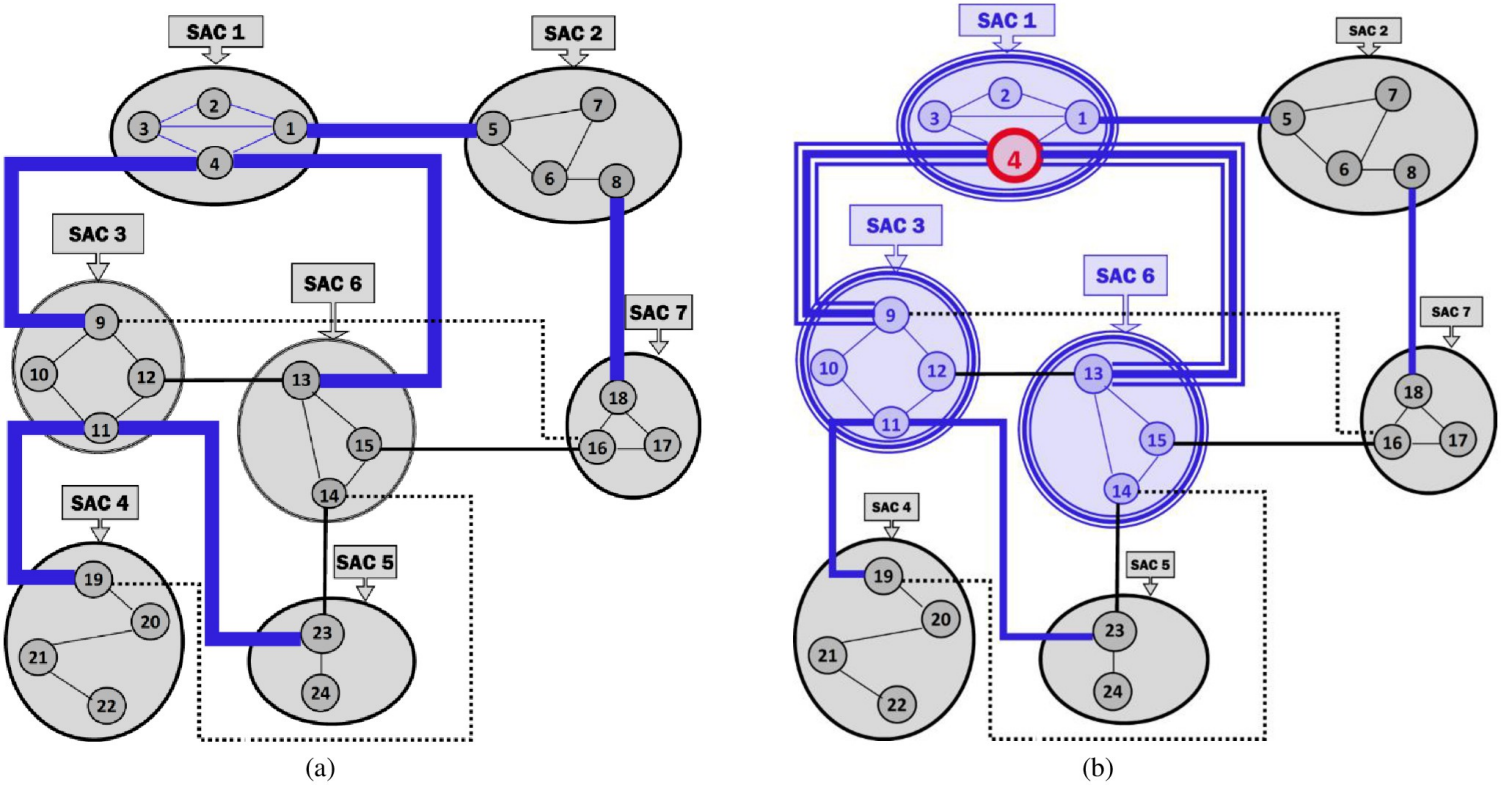
(a) The path of the MaxST in our running MRG example shown in Fig 20, and (b) the densest multi-profiled cross-SACs, to which the active user described in Example 9 belongs (i.e., the cross-SACs of SACs 3 and 6).

***Running Example 9***: After multiplying the Association Edges’ GIs scores in our running MRG example shown in Fig 20 by “-1” and then applying the Kruskal’s algorithm, we obtain the MaxST shown in Fig 21-a. Consider that an active user revealed that he belongs to SACs 3 and 6. As Fig 21-b shows, MKCSS 4 is the densest multi-profiled cross-SACs, to which the active user belongs (i.e., the cross-SACs of SACs 3 and 6). MKCSS 4 is located in the intersection of the MaxST’s paths that connects SACs 3 and 6.

## Experimental results

We aim at evaluating two features of our method ID_CC. The first one is ID_CC’s feature that detects the smallest cross-profiled cross-communities, to which an active user belongs. The second one is ID_CC feature that predicts missing implicit Association Edges in MRGs. We evaluated the first feature by comparing ID_CC with eight baseline models. Unfortunately, we could not find a comparable baseline model that predicts implicit Association Edges. Therefore, we evaluated the second feature as follows. We ran ID_CC against datasets that have no missing links. Then, we ran it against the same datasets but after removing some links. Finally, we compared the detected cross-communities before and after the links were removed.

### Baseline methods

We aim at evaluating ID_CC’s accuracy for detecting cross-communities by comparing it with eight baseline methods. These methods employ nodes’ attribute information as the basis for detecting communities. Below are brief descriptions of the eight methods:

1. ***DNMF***: It is a method proposed by Ye et al. [26] based on the nonnegative matrix factorization approach. DNMF can detect overlapping communities. It combines kernel regression and discriminative pseudo supervision techniques. The discrete community usership of a node is determined without post-processing.
2. ***LOCD***: It is a method proposed by Ni et al. [27] for detecting local overlapping communities. It employs bottom-up intermediary score maximization. Let *S* be a set of nodes that belong to at least two communities. The method selects a subset S′ ⊆ *S* as seed nodes. It identifies a community for each seed node. A node n′ is assigned to the same community of a seed node *n*, if the fuzzy relation between n′ and *n* is large enough.
3. ***CoRel***: It is a method proposed by Huang et al. [21] for building a seed-guided topical taxonomy. The method outputs a complete taxonomy from an input seed taxonomy and some corpus. It employs a module for relation transferring. After analysing seed taxonomy’s parent-child nodes and their relationships, the module transfers the learned information upwards and downwards to identify the topics and subtopics of the first layer. The method also employs a learning module to enhance the semantics of each node by determining its discriminative topical clusters.
4. ***RCF***: It is a method proposed by Guesmi et al. [28] that employs relational concept analysis for detecting communities from a heterogeneous information network. It generates a set of concept lattices iteratively. The method detects communities by navigating through the lattices.
5. ***Neo4j***: It is a graph database engine that adopts the Property Graph model [29]. A node can have multiple labels representing their roles in the graph. The method employs “patterns” and path-oriented graph procedures. It matches the variables of a query to a graph based on the graph’s patterns. Then, it outputs variables that represent the maximum number of labels relevant to the input variables of the query.
6. ***GKS*, *BRWS*, *and GLPS***: The three methods were proposed by Sharma et al. [30], as follows:
  - GKS is an expansion of the Katz approach [31], which adopts the concept of group accretion. It produces a score that reflects the degree into which an external actor matches a certain group. The score is the average proximity of the group’s actors to the external actor. The method enumerates the network’s paths using unsupervised path counting procedure.
  - BRWS is a method that adopts the group accretion concept. It uses semi-supervised learning and network alignment approaches to quantify the affinity among actors. By analysing cycles, the method determines the affinity of a group of users to an external actor. The method identifies the cycles that pass-through groups of nodes.
  - GLPS is a method that employs semi-supervised and hypergraph label propagation approaches. The method diffuses labels by random walks. After the random walks are stabilized, the final labels of the external nodes are considered as affinity scores to the given groups.
  - The main differences between the three methods are summarized as follows:
    - GKS assesses the affinity between an external actor and a group of users separately. On the other hand, BRWS assesses the affinity between an external actor and a subgroup within a group of users. The GKS method adopts an incremental accretion procedure that incrementally joins an external actor to an existing group. The BRWS method adopts a subgroup accretion procedure that considers the collaboration of external actors within a subset of an existing group.
    - The BRWS and GKS are based on paths and cycles over a *network of actors*. On the other hand, the GLPS method is based on paths on a *network of groups* (*NOG*) by adopting label propagation score, which is based on hypergraph structure.

The codes of the above methods are available as follows:

- The code of CoRel [21] is available at https://github.com/teapot123/CoRel.
- The code of LOCD [27] is available at https://github.com/ahollocou/multicom.
- The code for DNMF [26] is available at https://github.com/smartyfh/DNMF.
- As for GKS, GLPS, and BRWS, we used the same dataset and followed the same experimental setup employed for evaluating the three methods as described in Sharma et al. [30].

### Evaluation setup

We implemented ID_CC in Java, ran it in Intel(R) Core(TM) i7-6820HQ processor with 32 GB RAM and 2.70 GHz CPU under Windows 10 Pro. The demo application of ID_CC can be accessed through the following link: http://134.209.27.183/ (see Appendix for how the demo works).

Let *Տ* be the set of communities in a dataset. For each different subset *Ś* ⊂ *Տ*, we determined the subset *Ŋ* of nodes resulting from the intersection of the communities in *Ś* (i.e., their overlapping). We aim at using *Ŋ* as a ground-truth cross-community resulting from the overlapping of the subset *Ś*. That is, we evaluated the accuracy of each method for detecting the cross-community resulting from the overlapping of each *Ś* ⊂ *Տ* by comparing its results with the subset *Ŋ*. Intuitively, a method’s accuracy may decrease as the number of overleaped communities increases. Therefore, we also aim at evaluating a method’s accuracy stability as the number of overleaped communities increases. Towards this, we computed the accuracy of each method for detecting each subset *Ŋ* that results from the overlapping of *m different number* of communities in the subset *Ś*. Specifically, we considered *m* = | *Ś* | = 2, 3, 4, 5, and 6. The *m* number of communities are selected randomly. In the case of ID_CC, we considered *m* as the number of a hypothetical user’s revealed number of communities.

We evaluated the accuracies of the nine methods for detecting cross-communities in terms of Adjusted Rand Index (ARI) and F1-score measures. ARI computes the similarity of two clusters’ pair-wise comparisons. It is defined as:

ARI=((Index−Expectedindex)/(Maximumindex−Expectedindex)),

where:

$\text{Index}=\sum_{\text{ij}}\left(\begin{array}{l} {\text{n}}_{\text{ij}}\\ 2 \end{array}\right)$, $\text{Expected}\;\text{index}=\left\lfloor \sum_{\text{i}}\left(\begin{array}{l} {\text{a}}_{\text{i}}\\ 2 \end{array}\right)\;\sum_{\text{j}}\left(\begin{array}{l} {\text{b}}_{\text{j}}\\ \text{2} \end{array}\right)\right\rfloor /\left(\begin{array}{l} \text{n}\\ 2 \end{array}\right)$, and $\text{Max}\;\text{index}=\frac{1}{2}\;\left[\sum_{\text{i}}\left(\begin{array}{l} {\text{a}}_{\text{i}}\\ \text{2} \end{array}\right)+\sum_{\text{j}}\left(\begin{array}{l} {\text{b}}_{\text{j}}\\ \text{2} \end{array}\right)\right]$

We use ARI to assess the pair-wise similarities between the cross-communities detected by one of the methods and a corresponding ground-truth cross-community. F1- score is the harmonic average of recall and precision. It is defined as: F1-score = 2 (Recall x Precision) / (Recall + Precision).

### The accuracies of the methods for detecting the DBLP cross-communities

**Fig 22:**
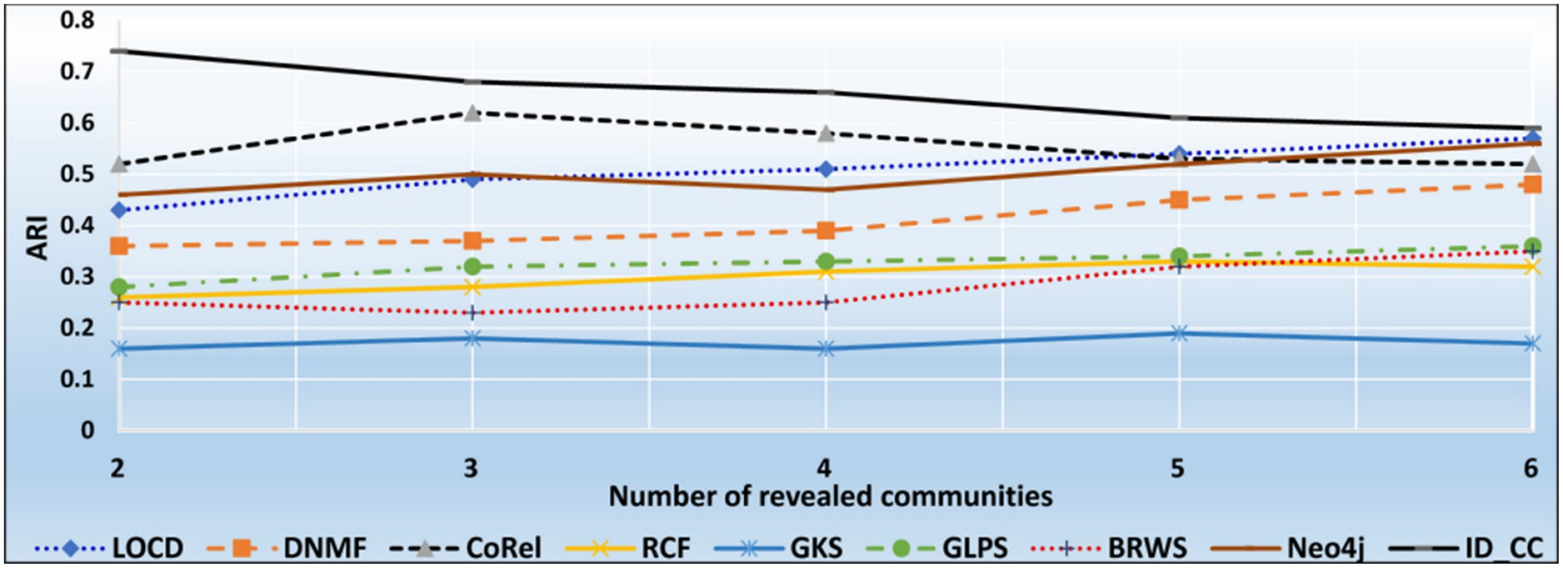
The accuracy of each method in terms of ARI for detecting the DBLP cross-communities resulted from the overlapping of *m* number of communities (*m* = 1, 2, …, 6).

**Fig 23:**
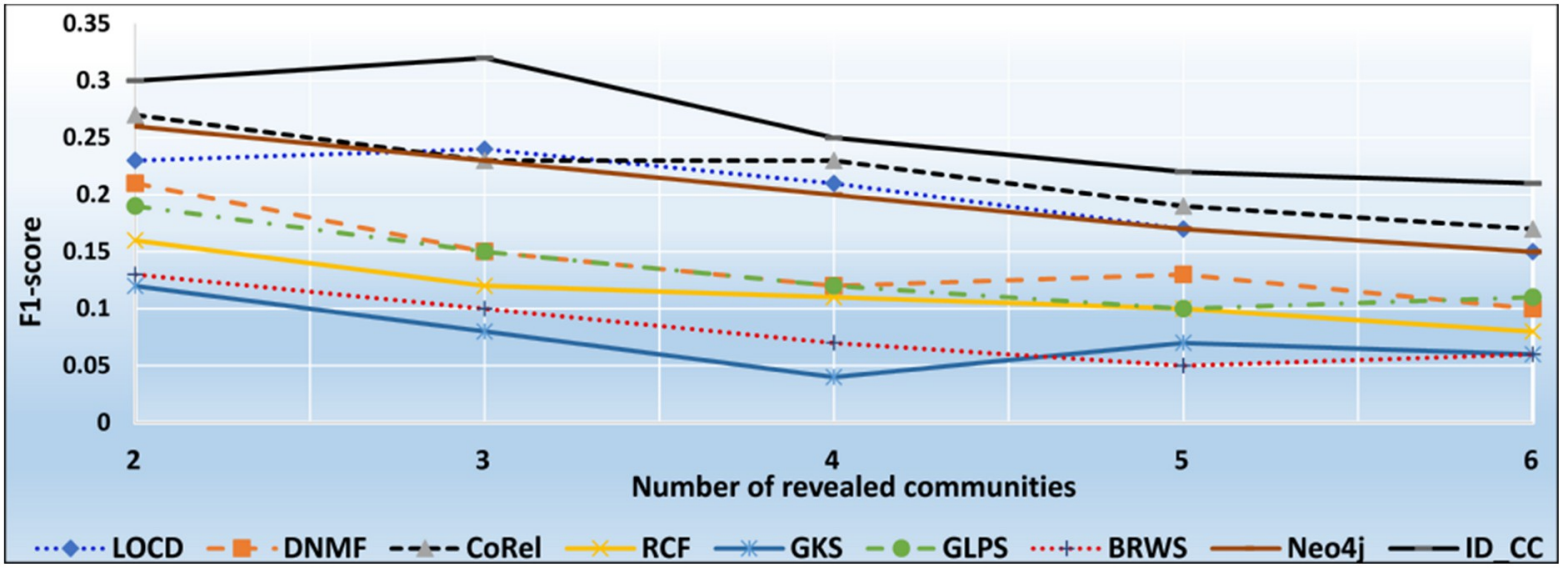
The accuracy of each method in terms of F1-score for detecting the DBLP cross-communities resulted from the overlapping of *m* number of communities (*m* = 1, 2, …, 6).

**Fig 24:**
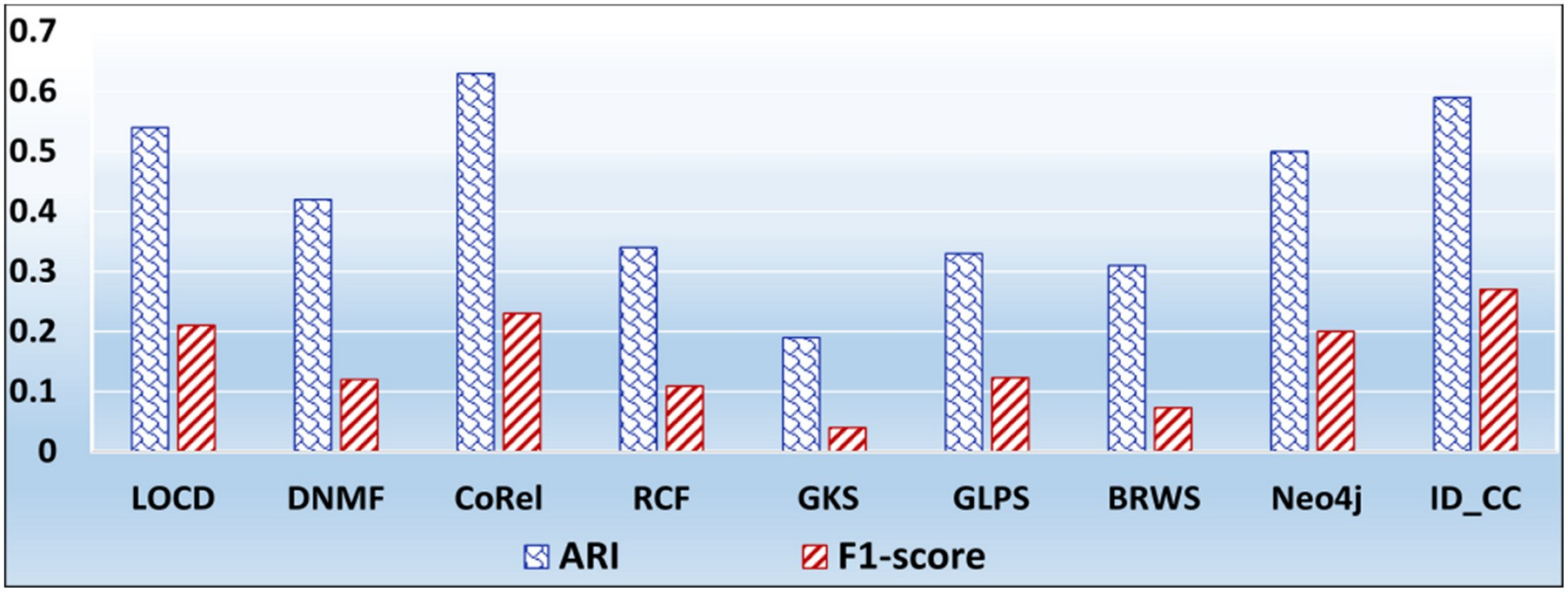
The overall average accuracy of each method for detecting the DBLP cross-communities in terms of ARI and F1-score.

The DBLP dataset is a collection of real-world ground-truth networks put together by the Stanford Network Analysis Project (SNAP) [32]. It includes a comprehensive list of research articles in computer science converted into co-authorship networks. These networks comprise 1,049,866 edges, 317,080 nodes, and 13,477 communities. Two nodes in the networks are linked by an edge, if the authors represented by the nodes published at least one research article together. A node represents an author, and an edge represents the number of common articles between two authors. Authors who published in a specific conference/journal constitutes a community. A group of authors, who participated in a same publication venue forms a ground-truth community. Figs 22 and 23 show the accuracy of each method in terms of ARI and F1-score, respectively, for detecting the DBLP cross-communities resulted from the overlapping of *m* number of communities (*m* = 1, 2, …, 6). Fig 24 shows the average accuracy of each method for determining the DBLP cross-communities in terms of ARI and F1-score.

To substantiate the findings and outcome of the previous tests, we also evaluated the methods by employing the same experimental setup and same dataset used for evaluating BRWS, GKS, and GLPS in [30]. This includes the following:

1. The same testing and training periods for the main splits. Splits are marked with fixed end years. Articles published in the years 2008 to 2010 were used for testing and articles published in the years 2004–2007 were used for training (see Table 2).
2. The same used dataset, which was the DBLP.
3. The same measures used, which were “Recall@N_top_ (IA)” and “Precision@N_top_ (IA)” for N_top_ = 100. These measures are defined as:
$$\text{Precision}@{\text{N}}_{\text{top}}\left(\text{IA}\right)=\left(\text{Number}\;\text{of}\;\text{correctly}\;\text{predicted}\;\text{groups}\;\text{using}\;\text{IA}\;\text{from}\;\text{top}–{\text{N}}_{\text{top}}\;\text{list}\right)/{\text{N}}_{\text{top}}$$
$$\text{Recall}@{\text{N}}_{\text{top}}\left(\text{IA}\right)=\left(\text{Number}\;\text{of}\;\text{correctly}\;\text{predicted}\;\text{collaborations}\;\text{from}\;\text{top}–{\text{N}}_{\text{top}}\;\text{list}\right)/\left(\text{Number}\;\text{of}\;\text{actual}\;\text{IA}\;\text{groups}\right)$$

Where, N_top_ is the N top sorted IA set, IA is the incremental accretion, and Top-N_top_ is the largest score in N top sorted IA set.

**Table 2:** Dataset division into training and testing splits.

Boundary Year	Split No.	Test	Train
2007	Main Split	2008–2010	2004–2007

**Fig 25:**
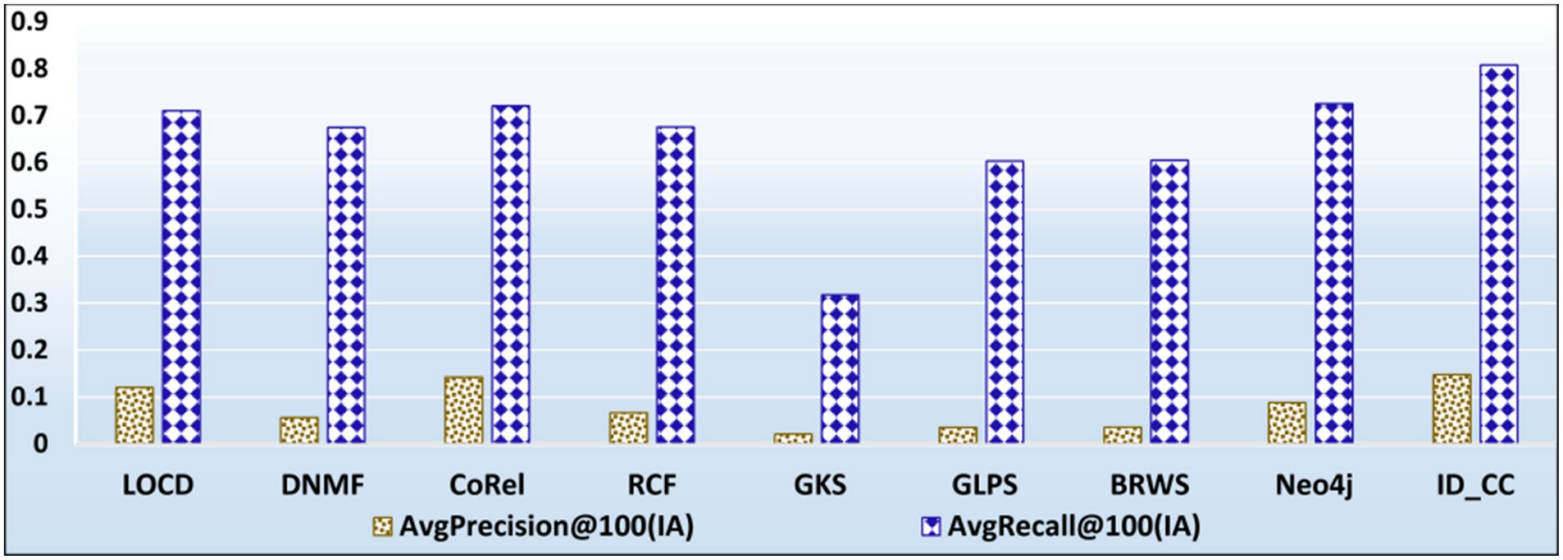
The prediction accuracies of the methods using the setup described in [7].

Fig 25 shows the accuracies of the nine methods based on the dataset division in Table 2. The accuracy values of BRWS, GLPS, and GKS shown in Fig 25 are the same ones presented in [2].

### The accuracies of the methods for detecting the Friendster cross-communities

**Fig 26:**
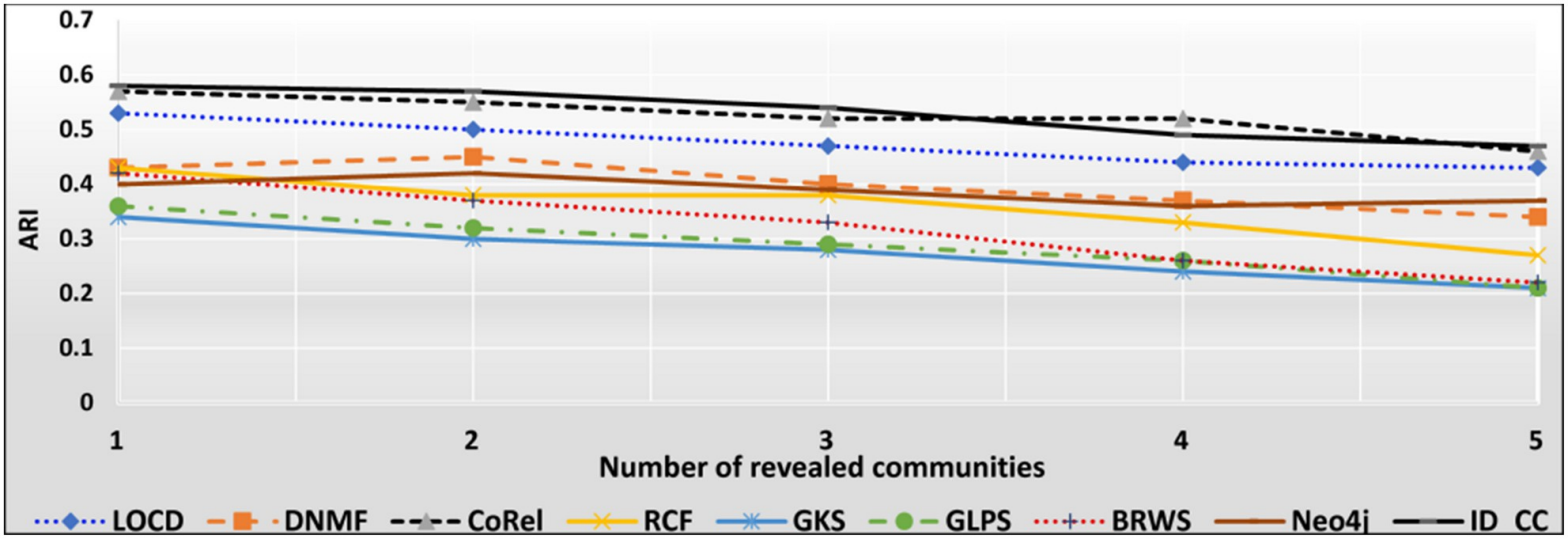
The accuracy of each method in terms of ARI for detecting the Friendster cross-communities resulted from the overlapping of *m* number of communities (*m* = 1, 2, …, 5).

**Fig 27:**
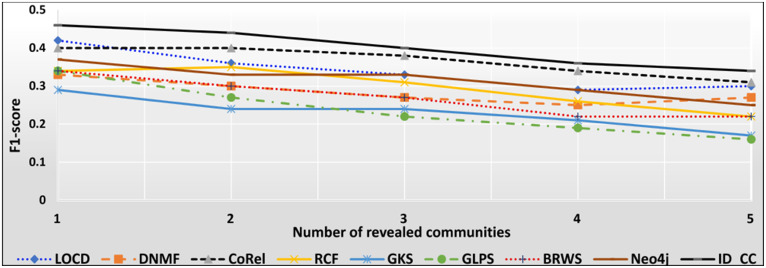
The accuracy of each method in terms of F1-score for detecting the Friendster cross-communities resulted from the overlapping of *m* number of communities (*m* = 1, 2, …, 5).

**Fig 28:**
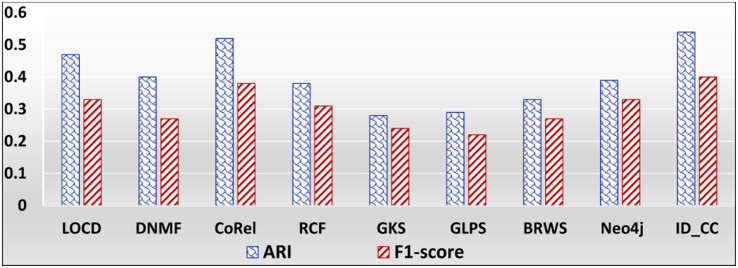
The overall average accuracy of each method for detecting the Friendster cross-communities in terms of ARI and F1-score.

The Friendster dataset is a collection of real-world ground-truth networks put together by SNAP [32]. The dataset consists of declared communities that belong to a social networking site and an on-line gaming network. Users of the sites declare friendships and construct groups. These declared user-defined groups are considered ground-truth communities. The networks comprise 957,154 communities, 1,806,067,135 edges, and 65,608,366 nodes. Figs 26 and 27 show the accuracy of each method in terms of ARI and F1-score, respectively, for detecting the Friendster cross-communities resulted from the overlapping of *k* number of communities (*k* = 1, 2, …, 5). Fig 28 shows the overall average accuracy of each method for determining the Friendster cross-communities in terms of ARI and F1-score.

### The accuracies of the methods for detecting the Facebook Social circles

**Fig 29:**
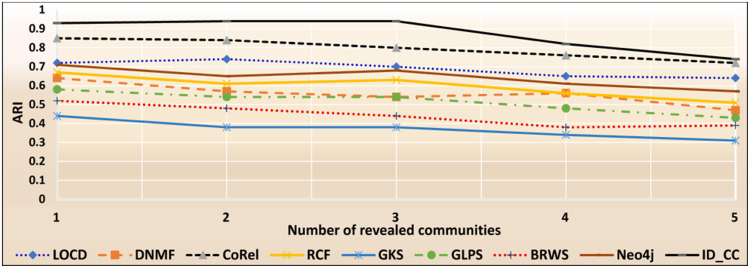
The accuracy of each method in terms of ARI for detecting the Facebook Social circles cross-communities resulted from the overlapping of *m* number of communities (*m* = 1, 2, …, 5).

**Fig 30:**
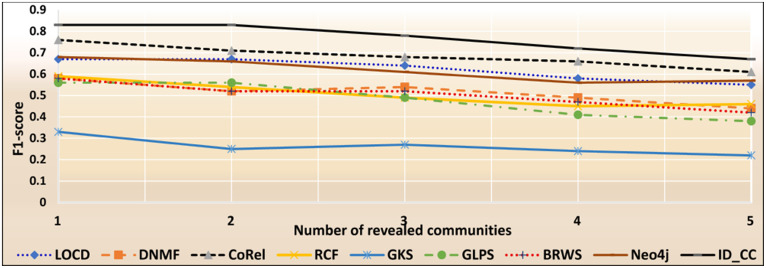
The accuracy of each method in terms of F1-score for detecting the Facebook Social circles cross-communities resulted from the overlapping of *m* number of communities (*m* = 1, 2, …, 5).

**Fig 31:**
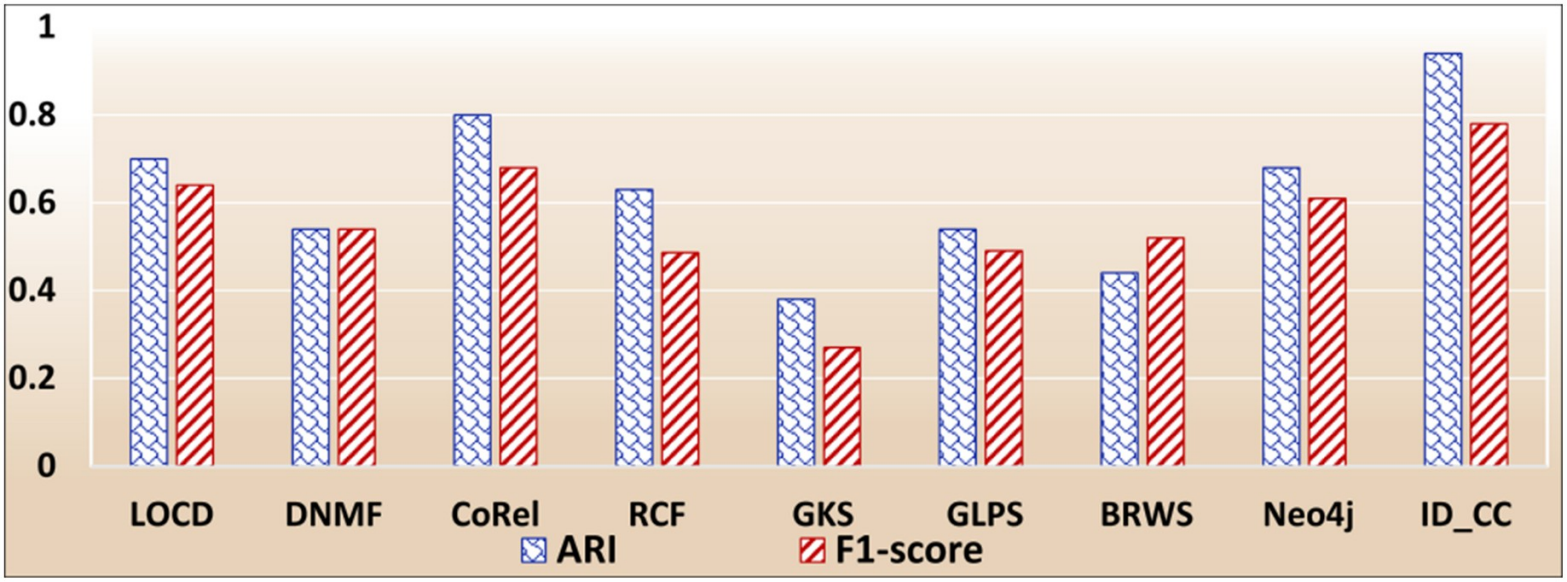
The overall average ARI and F1-score of each method for detecting the Facebook Social circles cross-communities.

The Facebook Social Circles dataset is a collection of real-world ground-truth networks put together by SNAP [32]. It was compiled by surveying 4039 Facebook users. It comprises a human social network and Ego networks. A node in the network depicts a user. Each node has its own Ego network, which contains the list of friends (i.e., circle) of the user. The Ego networks are built as follows: (1) each node is regarded as a focal node (i.e., an ego), (2) each other node (i.e., an alter) is connected to the focal node by an edge, if the two have a social relationship, and (3) each alter node has its own Ego network. The network comprises 88,234 edges. It also contains the profiles of users. The Facebook-internal ids are represented by new values, which makes it feasible for assessing whether two users have social relationship. In our evaluation, we considered each Ego network as a ground-truth community. Figs 29 and 30 show the accuracy of each method in terms of ARI and F1-score, respectively, for detecting the Facebook Social circles cross-communities resulted from the overlapping of *k* number of communities (*k* = 1, 2, …, 5). Fig 31 shows the overall average accuracy of each method for determining the Facebook Social circles cross-communities in terms of ARI and F1-score.

### Evaluating the effectiveness of ID_CC to uncover implicit Association Edges

**Fig 32:**
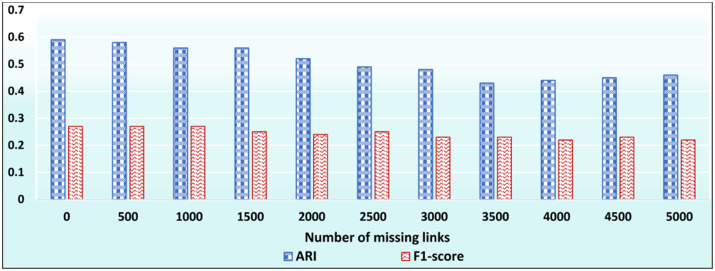
The accuracy of ID_CC in detecting the DBLP communities after identifying and reinstating the missing Association Edges.

**Fig 33:**
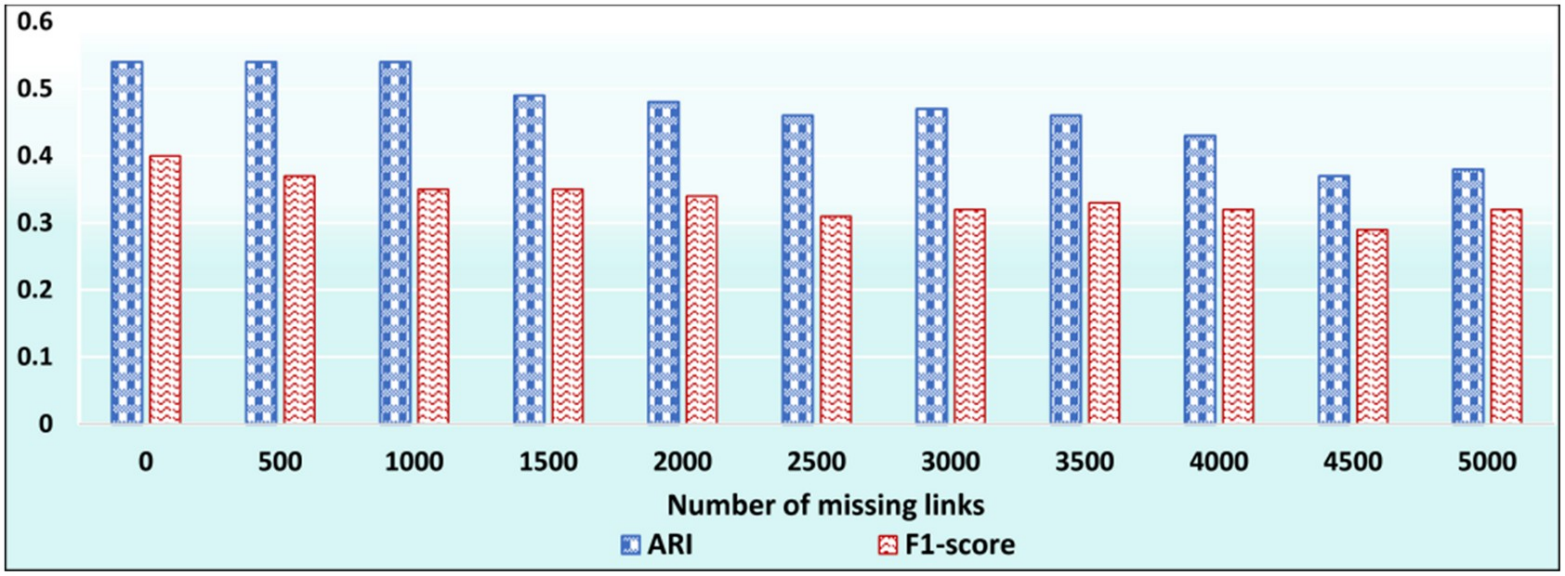
The accuracy of ID_CC in detecting the Friendster communities after identifying and reinstating missing Association Edges.

**Fig 34:**
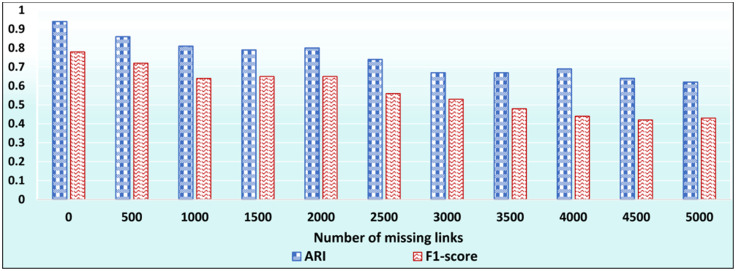
The accuracy of ID_CC in detecting the Facebook Social Circles communities after reinstating the missing Association Edges.

In this test, we aim at evaluating the effectiveness of ID_CC to uncover missing (implicit) Association Edges. First, we ran ID_CC to build the MRGs of the DBLP, Friendster, and Facebook Social Circles datasets. Then, we randomly removed 500 Association Edges from the MRGs and evaluated the accuracy of ID_CC in identifying and reinstating these edges. We then repeated the same procedure nine times. In each of the nine times, we increased the number of removed Association Edges by 500. That is, the number of removed edges were 500, 1000, …, 5000. ID_CC’s accuracy of identifying the missing Association Edges is assessed by its accuracy in detecting the original communities in the datasets after reinstating these edges. Figs 32, 33 and 34 show the accuracy of ID_CC in terms of ARI and F1-score for detecting the DBLP, Friendster, and Facebook Social Circles communities, respectively, after identifying and reinstating the missing Association Edges.

### Statistical test of significance

We used One-way ANOVA Test [33] to determine whether the differences between each method’s individual accuracy values in the tests described in the previous subsections are large enough to be statistically significant. ANOVA incorporates several statistical models, but we focused on the model that estimates the *variation within a group* to determine if the variation is large enough to be statistically significant. Since: (1) each group in our experiments represents the overlapping of different *k* number of communities (*k* = 1, 2, …, 5), and (2) it is known that the accuracy decreases as *k* increases, we did not focus on the model that estimates the *variation between groups*. However, we did consider the variation between groups in the context of computing F-Statistic to determine how large the variability between group means compared to the variability of the observations within the groups. Due to the specific nature of our experiments, we want the variation among a group to be small while F-Statistic to be large. Tables 3 and 4 show the results for the ARI and F-score tests respectively. As the tables show, the variations within groups are relatively small and the F-Statistics are relatively large for all methods except for GKS, BRWS, and GLPS.

**Table 3:** One way ANOVA table for the overall average values of the ARI tests.

		LOCD	DNMF	CoRel	RCF	GKS	GLPS	BRWS	Neo4j	ID_CC
**Within Groups**	**Sum of Square (SS)**	0.0078	0.0104	0.0008	0.0112	0.0222	0.0167	0.0181	0.0082	0.0065
	**Mean Square (MS)**	0.0008	0.0012	0.0008	0.0015	0.0022	0.0023	0.0019	0.0020	0.0007
**Between Groups**	**Sum of Square (SS)**	0.0255	0.0338	0.0277	0.0285	0.0318	0.0399	0.0515	0.0231	0.0361
	**Mean Square (MS)**	0.0064	0.0085	0.0069	0.0071	0.0086	0.0100	0.0129	0.0058	0.0083
	**F-Statistic**	9.1612	6.4647	9.4797	4.9175	2.4157	4.3595	7.0282	5.3125	21.253
	**p-value**	0.0364	0.0468	0.0380	0.0487	0.3421	0.0961	0.0102	0.2400	0.0461

**Table 4:** One way ANOVA table for the overall average values of the F1-score tests.

		LOCD	DNMF	CoRel	RCF	GKS	GLPS	BRWS	Neo4j	ID_CC
**Within Groups**	**Sum of Square (SS)**	0.0107	0.0125	0.0061	0.0127	0.0218	0.0160	0.0159	0.0038	0.0043
	**Mean Square (MS)**	0.0011	0.0012	0.0006	0.0012	0.0026	0.0021	0.0018	0.0004	0.0004
**Between Groups**	**Sum of Square (SS)**	0.0286	0.0226	0.0255	0.0265	0.0296	0.0370	0.0351	0.0279	0.0255
	**Mean Square (MS)**	0.0072	0.0056	0.0063	0.0073	0.0083	0.0093	0.0073	0.0070	0.0069
	**F-Statistic**	7.3960	6.4011	12.304	5.5758	2.2782	5.0041	4.6914	18.875	23.75
	**p-value**	0.0276	0.5567	0.0215	0.0310	0.1731	0.0518	0.0158	0.0124	0.0362

### Discussion of the results

#### Strengths of ID_CC

The experimental results demonstrated that ID_CC predicted cross-communities of nodes with multiple attributes with outstanding accuracy. The results showed that ID_CC outperformed the eight methods it was compared with. By observing the experimental results, we attribute the performance of ID_CC over the other methods, in general, to the combination of the following capabilities of ID_CC: (1) detecting granular multi-attributed cross-communities by analysing the hierarchical interrelationships and overlaps of single-attributed communities, (2) employing the novel concepts of MKCSS and MRG, which proved to produce effective graphical miniatures that depict communities and their ontological relationships, and (3) employing the novel concept of GI, which proved to be an effective mechanism for characterizing the global relative influences and interaction roles of Association Edges in MRGs.

**Fig 35:**
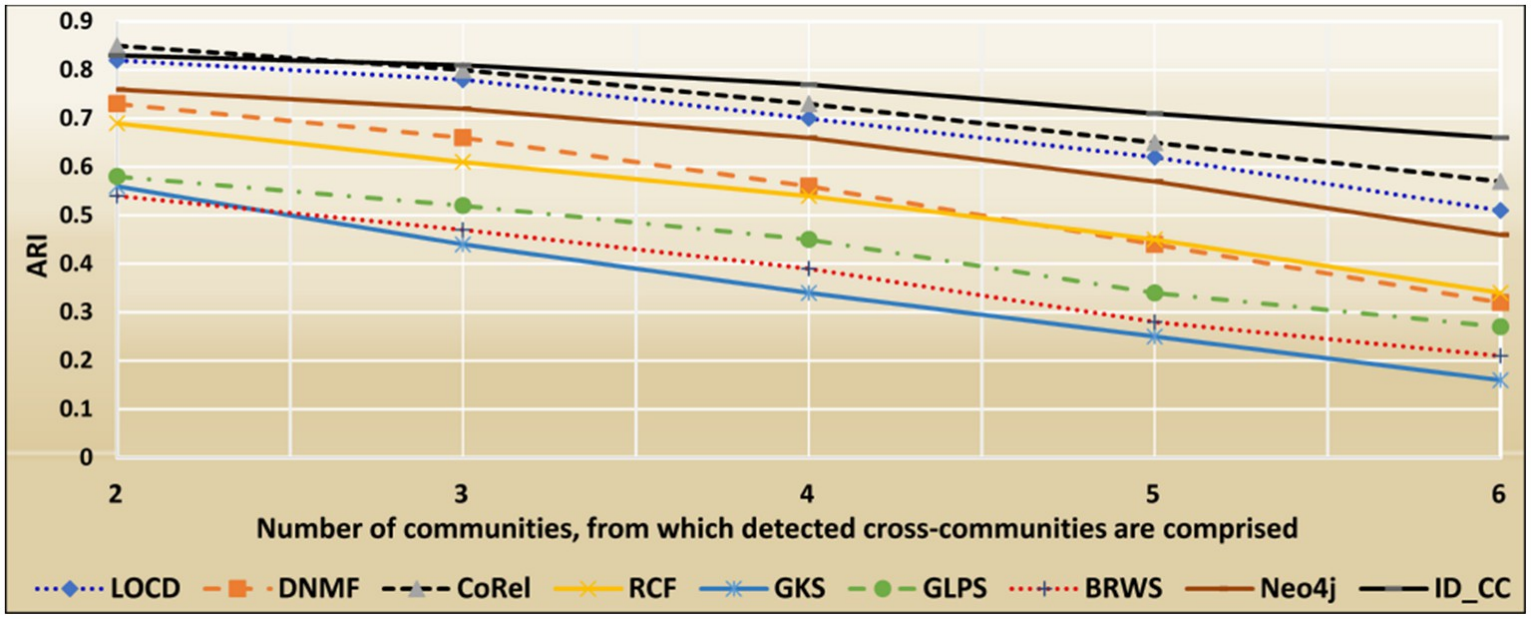
The average ARI for each group of detected cross-communities, where each group contains cross-communities comprised of the same number of single-attributed communities.

We observed from the experimental results that the accuracy of each of the nine methods decreases as the number of single-attributed communities, from which its detected cross-communities are constructed increases. However, ID_CC’s accuracy decreases in much smaller rate than the other eight methods as the number of communities, from which detected cross-communities are comprised increases. To confirm the above, we classified the cross-communities detected by each method into groups. Each group contains detected cross-communities comprised of the same number of single-attributed communities. We then computed the overall average ARI for each set as depicted in Fig 35. As Fig 35 shows, the decrease rate of ID_CC’s accuracy is lower than the other methods as the number of communities, from which detected cross-communities are comprised increases.

We attribute the above, mainly, to ID_CC’s concepts of MKCSS and MRG, which helped in locating cross-nodes regardless of the number of communities, to which these nodes belong. As a community grows smaller, its interests become more specific, which is manifested in the users’ profiles of the Facebook Social circles dataset. Finally, the constant enhancement of MRG contributed further to the performance of ID_CC. This is because, every time ID_CC detected a cross-community, it enhanced the MRG accordingly by incorporating newly detected missing Association Edges.

#### Limitations of ID_CC

**Fig 36:**
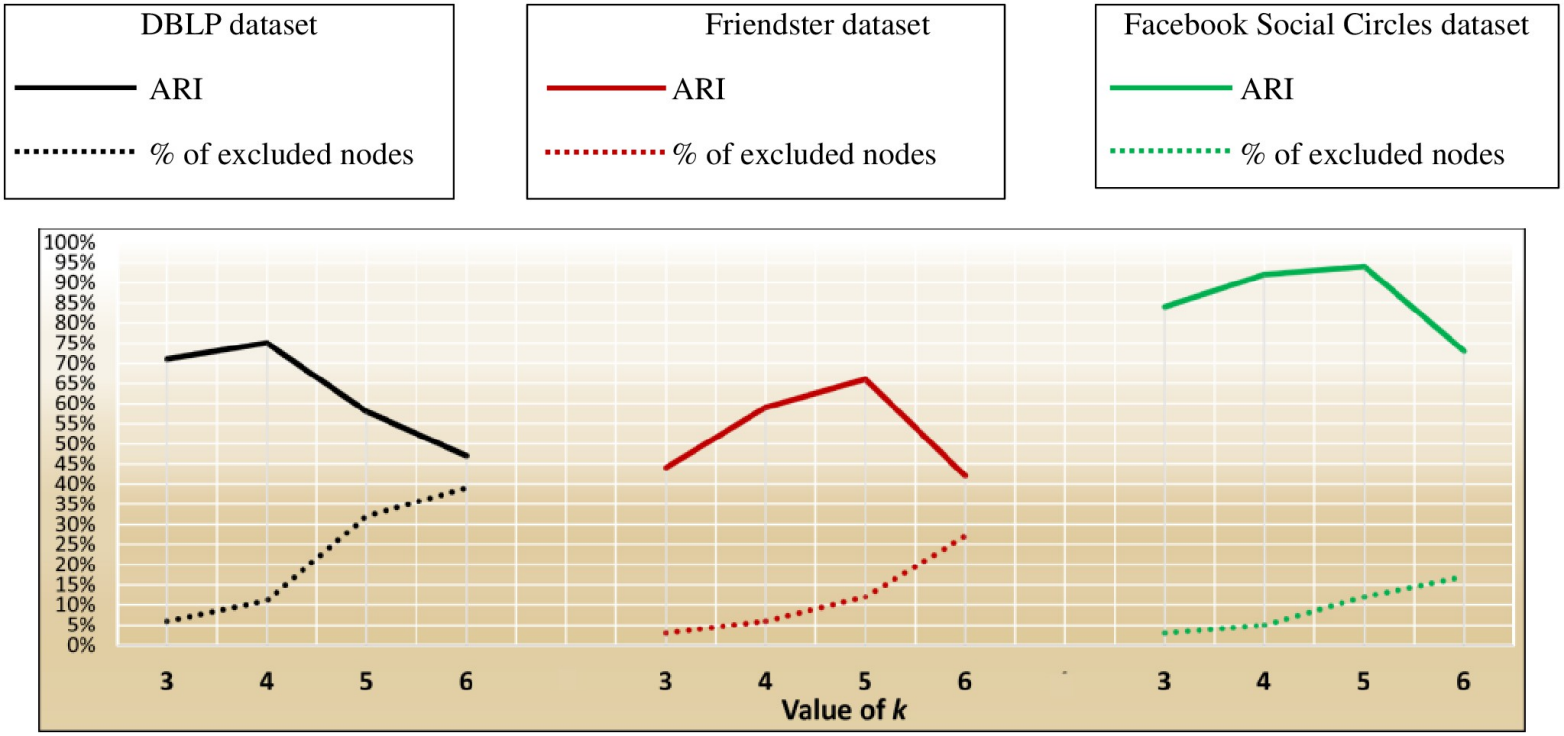
ARI and the percentage of nodes that are non-member of any MKCSS under different values of *k* in the range 3–6.

We observed from the experimental results that the value selected for k in k-clique had an impact on the accuracy of IC_CC. That is, ID_CC’s accuracy was to some degree k-dependent. We observed that its accuracy kept improving as the value of *k* increased up to a certain value and then it kept declining thereafter. After investigating this phenomenon, we inferred the following: (1) increasing the value of k leads to enhancing MKCSS (as k increases the MKCSS keeps retaining only strongly associated nodes and discarding other nodes), and (2) as the value of k increases, the percentage of nodes that become non-member of any MKCSS increases (as k increases, the degree into which nodes are constrained and retained within the boundaries of their MKCSSs decrease). Specifically, we observed that the accuracy kept improving (inference (1)) until a certain value of k where the percentage of non-member nodes became large enough that resulted in degrading the accuracy (inference (2)). That is, the accuracy degrades as the percentage of non-member nodes increases. To confirm the above, we varied the value of k in the range 3–6. Under each different value of k, we performed the following: (1) computed the ARI of the results, and (2) computed the percentage of nodes that are non-member of any MKCSS. Fig 36 depicts the findings of the test. As the figure shows, the accuracy of ID_CC kept improving until a certain value of k and then kept degrading. We will investigate approaches for overcoming this limitation in a future work. We will investigate a mechanism that helps in predicting the optimum value of *k*.

#### Strengths and Limitations of the methods proposed by Sharma et al. [30] (i.e., GKS, BRWS, and GLPS)

We observed that GKS inferred with acceptable accuracy cross-communities resulted from the overlapping of communities with rather high degree of common attribute homogeneity. However, the accuracy of its detection was poor for most of the cross-communities resulted from the overlapping of communities that exhibited high attribute heterogeneity. We attribute this limitation to GKS’s Katz score, which proved to be very ineffective in identifying the significant features of attributes, which would be employed for measuring the similarity between communities. The score is ineffective for determining the features in the profiles of communities that dictate their relationships. We also observed that the Katz score is sensitive to small differences between the profiles of an ego and an ‘alter’ in the Facebook Social Circle dataset. A small change between the profiles of an alter and an ego resulted in a large change in the Katz score. This was evident in scenarios where two alters have minor changes in their profiles, yet they achieved significantly different Katz scores. As a result, the score may mistakenly consider an alter as related to an ego.

We observed that the BRWS method inferred with acceptable accuracy most of the cross-communities that exhibited too many *direct links* between internal and external actors. However, the method had an inconsistency in measuring the affinity between external and internal target actors through *indirect links*. This is due to the fact that the bi-random walk technique adopted by the BRWS method has the limitation of random fluctuations when measuring the affinities between different external and internal actors through the indirect links connecting them. As a result, the method may produce misleading affinity scores.

We observed that GLPS inferred with good accuracy most of the cross-communities composed of nodes that have loose dependencies with one another. However, the hypergraph-based clustering technique employed by GLPS can cause dependencies between nodes. This resulted in many independent nodes. Moving these nodes to a cross-community required other dependent nodes to be moved with them, which is incorrect. Moreover, GLPS detected cross-communities that did not conform to tighter balancing constraints, which caused hyperedge to span several cross-communities. This cause the sizes of cross-communities to be incorrectly increased.

#### Strengths and Limitations of the RCF Method

The experimental results revealed that RCF was successful in building global sequences of context sets and their corresponding sequences of lattice sets from the datasets. This is attributed to the Concept Analysis technique adopted by RCF, which helped it in successfully converting the links between a dataset’s objects into attributes and inferring a set of lattices whose concepts are linked by relations. The method did so in relations consisted of a small to moderate number of instances of the relations. However, the method was not successful in inferring sets of lattices, whose concepts are linked by relations consisted of many instances of the relations. The key limitation of RCF is that it considers only a limited number of quantifiers. It performed miserably in relations required a combination of quantifiers not in the considered set.

#### Strengths and Limitations of the Neo4j Method

The experimental results revealed that the property graph technique employed by Neo4j was effective in scenarios where edges and nodes possess different types of meta-information. We observed that the Neo4j’s pattern matching of nodes while traversing a graph contributed significantly to the accuracy of cross-communities inferred by the method. This is due to Neo4j’s effective path-oriented queries and Cypher’s declarative graph query language. The method proved to be effective in clustering complex networks (e.g., with many levels) into accurate cross-communities. This is because the ecosystem and its associated functionality on top of Neo4j helped it in storing infinite levels of community overlaps. By analyzing the experimental result, we deduced the following limitations of Neo4j: (1) it allowed only one label per edge and one value per attribute property whereas some datasets have multiple labels and values, (2) Cypher adopts no-repeated-edge semantics, and (3) it had indexing limitations, especially for edges annotated with attribute terms.

#### Strengths and Limitations of the DNMF Method

The accuracy results achieved by DNMF were satisfactory overall. After investigating the results, we deduced this performance to DNMF’s adoption of the mutual guidance of the following two information types: (1) guidance information learnt through a unified manner: pseudo supervision module (which adopts unsupervised procedure for uncovering discriminative information), and (2) guidance information learnt through community memberships. By observing the experimental results, we found that DNMF obtained good results when the tradeoff parameter was not assigned large values. The method did not achieve good results when: (a) the size of values assigned to it was large, (b) the number of overlapping memberships was large, or (c) the number of overlapping nodes was large. The method exhibited good execution time. We attribute this to the algorithm employed by the method, which decomposes the objective function into independent subproblems without the need for post-processing.

#### Strengths and Limitations of the LOCD Method

By analyzing the experimental results, we found that the accuracy of LOCD kept improving as the number of seed nodes selected by the method increased. This is because as the number of seed nodes increases, the number of nodes bordering the seed nodes and share characteristics similar to the detected communities increases. We also found that LOCD’s number of accurately selected seed nodes increases as the fuzzy relation threshold increases. Specifically, we found that LOCD inclined to obtain good results after setting the fuzzy relation threshold to at least 0.87 (we used this threshold in the evaluations of LOCD). The method outperformed most of the other methods in inferring cross-communities that contain some nodes belonging to disconnected subnetworks. The Facebook Social Circles and Friendster datasets exhibited many of such disconnected subnetworks. We attribute this to the local community detection technique employed by LOCD, which helps in overcoming the problem of missing global information in disconnected subnetworks. The major limitation of LOCD stems from the fact that its detection accuracy is highly dependent on parameters’ setting.

#### Strengths and Limitations of the CoRel Method

Overall, CoRel achieved outstanding accuracy results. We attribute this, mainly, to the methodology it employs for constructing taxonomies to detect the related terms of each concept. By observing the experimental results, we found that CoRel successfully inferred the related terms associated with a community’s profile/property terms for many concepts. It extracted distinctive terms for many network nodes effectively. We deduced that the co-clustering procedure adopted by the method to ignore inconsistent subtopics played a significant role in its outstanding performance. The major limitation of CoRel stems from its enriching procedure, which did not effectively enforce term distinctiveness in a number of networks.

## Conclusions

The most important types of such multi-profiled cross-communities are the *densest* holonic ones with *various* adaptive *multi-social profiles*, because they exhibit many interesting properties. Unfortunately, methods that stress the detection of granular multi-profiled cross-communities have been under-researched. Most current methods detect multi-profiled communities without consideration to their granularities. To overcome this, we introduced in this paper a novel methodology for detecting the smallest and most granular multi-profiled cross-community, to which an active user belongs. The methodology is implemented in a system called ID_CC. The proposed system considers all cross-profiles that come to existence from the interrelations between overlapped social profiles (both known and implicitly inferred overlaps). It employs the novel concepts of MKCSS and MRG, which proved to produce effective graphical miniatures of communities and their ontological relationships. It also employs the novel concept of GI, which proved to be an effective mechanism for characterizing the *global* relative influence and interaction role of Association Edges in networks.

There are always new users wishing to join cross-communities that match their own social traits. ID_CC detects cross-community in such a way that it matches a new user’s own social traits. The larger the number of inferred user’s communities, the denser and more specific is the multi-profiled cross-community identified by the system for the user. Towards this, ID_CC implicitly infers an active user’s undeclared and unknown communities that match his own social traits using novel techniques. It detects cross-communities by analysing hierarchically overlapped social profiles to infer all cross-profiles that come to existence from the interrelations between the communities. It detects the densest multi-profiled cross-communities from heterogeneous social networks.

To the best of our knowledge, this is the first work that: (1) analyses hierarchically overlapped social profiles to detect the *densest and most granular* multi-profiled cross-communities, to which an active user belongs, (2) assesses the binary and global influences of the links connecting community nodes using novel mechanisms, (3) infers missing links prior to detecting cross-communities using novel mechanisms, and (4) employs novel graphical miniatures that depict communities and their ontological relationships.

We evaluated ID_CC by comparing it experimentally with the following eight methods: CoRel [21], LOCD [27], DNMF [26], RCF [28], Neo4j [29], GKS [30], GLPS [30], and BRWS [30]. The experimental results demonstrated that ID_CC predicted cross-communities of nodes with multiple attributes with outstanding accuracy. The results showed that the accuracy of each of the nine methods decreased as the number of attributes in the method’s detected cross-communities increased. However, ID_CC’s accuracy decreased in much smaller rate than the other eight methods as the number of communities, from which detected cross-communities are comprised increased. It was evident that analysing the *hierarchical* interrelationships of single-attributed communities using the concepts of MKCSS, MRG, and GI played a considerable role in the quality of cross-communities detected by ID_CC. We observed from the results that the value selected for *k* in *k*-clique had an impact on the accuracy of IC_CC. That is, ID_CC’s accuracy was to some degree *k*-dependent. We will investigate approaches for overcoming this limitation in a future work.

## Supporting information

S1 Appendix (DOCX)
