# Supplementary material for: Detecting implicit cross-communities to which an active user belongs
Source: PLoS One. 2022 Apr 19;17(4):e0264771. doi: 10.1371/journal.pone.0264771 (PMC9017956; doi:10.1371/journal.pone.0264771)
Supplement: S1 Appendix — (DOCX) [file pone.0264771.s001.docx]

**Appendix**

**Description of the demo application (** [**http://134.209.27.183/**](http://134.209.27.183/) **)**

The demo application of our system ID_CC can be accessed through the following link *(please open the link in Google Chrome)*: [**http://134.209.27.183/**](http://www.diseasesusceptibility.com)

**The demo application works as follows:**

The demo is pre-uploaded with messages associated with eight users. The GUI of the demo contains three buttons labelled “Demo Messages”, “Detect Smallest Cross-Community”, and “Add User and Messages”. Below is a description of each button:

Button “**Demo Messages**”: By clicking this button, the demo performs the following: (a) displays the set of prominent keywords extracted from the messages associated with each of the eight users, (b) constructs the SACs based on the similarity of prominent keywords (users, whose prominent keywords are similar constitute a SAC), and (c) constructs MKCSSs based on the 4-clique modelling.

Button “**Detect Smallest Cross-Community**”: By clicking this button, the demo detects and displays the densest cross-SACs.

Button “**Add User and Messages**”: This button is associated with the demo’s functionality, which infers the densest cross-SACs, to which an active user belongs. By clicking this button, a window appears where the new user’s information needs to be entered. The information includes the new user’s name and the messages associated with him/her. Then, and after clicking “Add”, the new user’s information will be uploaded. Finally, by clicking on the button “Detect Smallest Cross-Community”, the demo will display the lists of cross-SACs, including the cross-SAC of the new user, whose information was just entered.
